# Assessment of domestic pig–bushpig (*Potamochoerus larvatus*) interactions through local knowledge in rural areas of Madagascar

**DOI:** 10.1038/s41598-024-67208-1

**Published:** 2024-07-15

**Authors:** Rianja Rakotoarivony, Daouda Kassie, Alpha Andriamahefa, Diana Andria-Mananjara, Mihaja Rakotoarinoro, Herilantonirina Solotiana Ramaroson, Modestine Raliniaina, Miatrana Rasamoelina, Jose Pablo Gomez-Vazquez, Ferran Jori

**Affiliations:** 1 Joint Research Unit-Animal-Health-Territories-Risks-Ecosystems (UMR ASTRE), CIRAD, Campus International de Baillarguet, Montpellier, France; 2 National Centre for Applied Research in Rural Development- Department of Zootechnical Veterinary and Fish Farming Research (FOFIFA-DRZVP), Antananarivo, Madagascar; 3 Institut Pasteur de Madagascar, Antananarivo, Madagascar; 4 Center for animal disease modeling and surveillance (CADMS), Department of Veterinary Medicine and Epidemiology, University of California, Davis, USA; 5 Department of Zoology and Entomology, University of Pretoria, Pretoria, South Africa

**Keywords:** Ecological epidemiology, Epidemiology

## Abstract

In many parts of the world, domestic and wild animal populations interact at the interface between natural and agricultural ecosystems. Introduced with the first inhabitants arriving from eastern Africa, the bushpig (*Potamochoerus larvatus*) is the largest living terrestrial mammal in Madagascar. Bushpigs are regularly reported close to human settlements where they damage crops and gardens. As domestic pigs are often raised in free-ranging conditions around the villages, bushpigs and domestic pigs can interact leading to the transmission and circulation of shared swine pathogens that impact both animal and human health. In this study, we characterized the socio-ecological context of bushpig–domestic pig interactions in two different regions of western Madagascar. We conducted participatory mapping sessions and focus group interviews with 65 hunters, 80 pig farmers and 96 crop farmers in 20 fokontany, the smallest administrative unit in Madagascar. After discussing with participants, we gathered information about the spatialization of interactions and their potential geographical drivers. We explored data by performing multiple correspondence analysis and hierarchical clustering on principal components. Based on the reported occurrence or absence of bushpig-domestic pig interactions we were able to classify areas with high or intermediate levels of interactions or no interactions at all. Interactions between the two pig species were reported in only 25% of the fokontany assessed. Even though both suid species were attracted to fruit trees, crops, and water sources, only indirect interactions in those spots were reported. Direct interactions were reported in 10% of cases and referred to interspecific sexual and/or agonistic behavior. The participatory methods used to acquire local knowledge about natural events were confirmed as valuable, low-cost exploratory methods to characterize areas with wild-domestic animal interactions. The results of this study will help plan future studies to characterize the interface between the two species from an ecological or epidemiological perspective using more sensitive and sophisticated ecological approaches.

## Introduction

Interactions between domestic animals and wildlife have been occurring since domestication began about 12,000 years ago^1^. Wildlife-livestock interfaces are the physical spaces where wildlife and livestock ranges overlap and the species have the opportunity to interact^2,3^. Such interfaces are dynamic^4^ and the way livestock is raised can facilitate the occurrence of interactions with wildlife, particularly when domestic animals are raised in free-ranging conditions. In recent years, several studies focusing on wildlife-livestock interfaces have been undertaken in multiple areas of interest because of their implications in terms of ecological impact or disease transmission^5,6^.

Wild pigs are considered in many parts of the world to pose a real risk of spreading several human-derived pathogens, to other wildlife, and to domestic animals^7–9^. In this context, interactions between wild and domestic pigs and the possible resulting bidirectional transmission of African swine fever (ASF) virus have attracted increasing attention. In many regions, wild populations of suids play a key role in the maintenance and spread of this disease which is becoming a global pig pandemic^10–15^. The disease has an enormous economic impact on smallholders and emerging farmers, affecting the incomes of poor livelihoods who rely on pig farming for their savings and food security^16,17^.

To confirm that wildlife-livestock interfaces are spaces where wild and domestic species interact, we need to define the nature of their interactions (e.g., overlapping home ranges, contact, competition, predation) in a spatiotemporal matrix^18^, which in this study, concerns two species of suids, the domestic pig (*Sus scrofa domesticus*) and the local bushpig (*Potamochoerus larvatus*). Bushpigs are distributed across East and Southern Africa and in Madagascar. Due to their elusive and nocturnal habits, knowledge about their ecology and behavior is very limited. In Madagascar, bushpigs are not an endemic species. They are believed to have been introduced from eastern Africa by the first inhabitants approximately 2000 years ago^19^ and since the extinction of Malagasy megafauna, bushpigs are one of the largest terrestrial mammals in the Island. The species is classified by Malagasy law as an agricultural pest and can be hunted all year round^20^. Given its capacity to adapt to different ecological settings, bushpigs are assumed to be present in all types of Malagasy forest, including intact and distributed moist forest and can come close to human settlements where domestic pigs are present^21^.

Pig farming in Madagascar ranks second (in value) and third (in number) among livestock species which also include cattle, goats, sheep and poultry^22^. Pig farming is widespread throughout the island and is a source of income for many households. Work is family-based, rarely salaried, and food costs are high for poor rural households. Most domestic pigs are raised in free-ranging mode in rural areas of Madagascar, a situation also found in other African countries^23^.

Several ecological methods are used to collect data on interactions at the wildlife-livestock interface. These methods vary in their ability to quantify or simply detect potential interactions between different species or individuals^24–26^. Among these approaches, one that has been widely used in various world contexts is the collection of local knowledge. This method is considered practical, efficient, and appropriate for gathering initial information in a timely manner to understand and explore the occurrence of unusual ecological events such as animal interactions of which local stakeholders may be privileged observers^8,27–29^. Anthropological studies have long been conducted among traditional pastoralists, particularly in Africa, and have led to a better understanding of the relationship between farmers and their livestock and to the documentation of traditional remedies^30^. Participatory mapping is a crucial step in identifying the interfaces between domestic and wild animals^31^ that has been less widely applied up to now. Indigenous knowledge derived from the experience of herding animals over long periods of time enables pastoralists to define the spaces frequented by their animals and their behavior^32^. The fundamental spatial relationship between local communities and the natural environment in which they live is often poorly understood by government planners and/or policy makers^33^.

The study of interspecific interactions has been an important topic in applied sciences in the last two decades^34^. However, these interactions are diverse and complex, leading to the development and adaptation of different approaches, which have been borrowed from different disciplines to address the complexity of the problem^18^.

The aim of the present study is to use local knowledge to characterize the socio-ecological context in which interactions occur between domestic pigs and bushpigs in rural areas of Madagascar. These interactions often reported in many parts of the world, have never investigated, and analyzed in detail despite their important implications for the transmission of pathogens to domestic pigs and their management. To achieve this goal, we tested a combination of participatory methods including participatory mapping and focus group discussions among local rural communities in our study area. The data collected was analyzed to identify any ecological factors and farming practices that could influence the occurrence of such interactions.

## Materials and methods

### Choice and description of study sites

**Figure 1:**
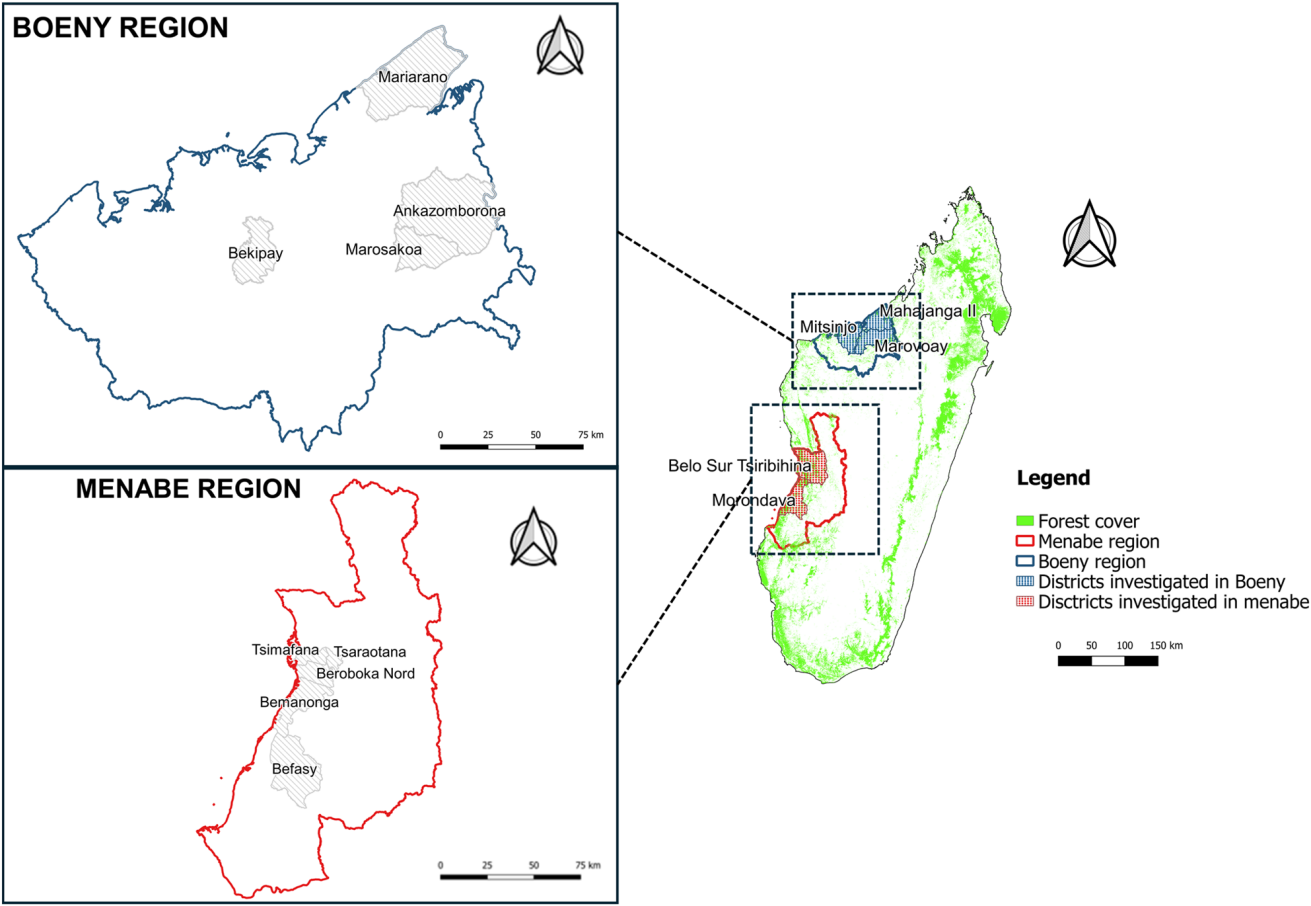
Map of study areas in Boeny and Menabe regions, 2021, Madagascar. Top left: Communes of Boeny region, bottom left: Communes of Menabe region.

The study was carried out in two different regions of Madagascar located in the central west and northwestern parts of the island (Fig. 1).

In each region, we identified several districts based on the occurrence of free-range pig farming, the abundance of forest cover particularly around protected areas and the presence of crops. This included the districts of Morondava, Belo Sur Tsiribihina in the region of Menabe and in the districts of Mahajanga II, Marovoay and Mitsinjo in the region of Boeny. Previous research conducted in these locations indicated the presence of free ranging pigs and bushpigs^21,35–38^. In addition, interactions between bushpigs and domestic pigs were also suspected to occur in those districts based on the literature and available data^20,39^.

The climate of Boeny region is sub-humid to dry^40^, annual rainfall ranges from 600 to 1500 mm and occurs mainly in summer, i.e. from January to April. The vegetation cover consists of dry deciduous forests, forests in various stages of degradation, mangroves and palm savanna. The landscape is physiognomically diverse, depending on rainfall and ranges from forest to impenetrable thickets to bushland and low scrub. With the exception of vegetation growing along the rivers, it is largely deciduous^41^. As the population is predominantly Sakalava, most pigs are raised in free ranging conditions and feed on household or other waste. The study sites in Boeny region included several protected areas, the Mahavavy Kinkony Wetland Complex in the center (302,000 ha), the Mariarano Special Reserve in the north (41,006 ha), and the Ankarafantsika National Park (130,000 ha) in the east.

In Menabe region, where the climate is sub-arid to sub-humid climates with rainfall ranging from 600 to 1200 mm per year (mean 795 mm)^41^, two types of vegetation predominate, mangroves and dry forests. The dry forests are known for their distinctive features such as Didiereaceae, Euphorbia thickets and sclerophyllous clear forests. Secondary formations of the tree savanna type with heliophilous perennial grasses are used as pasture for cattle and goats raised using both extensive and semi-intensive methods. Pig farming concerns around 96,500 animals^42^, mainly distributed in the districts of Morondava, Belo-Sur-Tsiribihina and Mahabo. The study sites in Menabe region are situated in close proximity to two important protected areas: Menabe Antimena Protected Area covers an area of 210,312 ha in the north, and the Kirindy Mitea National Park covers 625,000 ha in the south.

In these regions, we deliberately tried to identify locations that hosted the concomitant presence of the two species and hence the potential occurrence of interactions between bushpigs and domestic pigs. To that end, 20 fokontany (the smallest administrative unit in Madagascar) were selected after consulting authorized veterinarians and managers of the protected area in two regions where the occurrence of interaction between bushpigs and domestic pigs had been reported (Table 1). Logistical criteria such as accessibility, security, and the availability of contacts were also taken into consideration in the selection of the study areas. Each fokontany was assigned a unique identification number from 1 to 20. Specifically, the study was conducted in 16 administrative units (i.e., fokontany) in the Menabe region (median of population in each fokontany 1374; median of surface area is 96.4 km^2^). In the Boeny region, the study area included a total of 4 fokontany, including 2 in the west and 2 in the east (median of population in each fokontany 1757; median of fokontany area 183.2 km^2^).

**Table 1:** Characteristics of each fokontany in Madagascar included in this study (population size as of 2021, derived from Third General Census of Population and Housing in Madagascar).

Region	District	Rural commune	Fokontany identifier	Fokontany area (km^2^)	Population
Boeny	Mitsinjo	Bekipay	17	193.3	2482
			5	173.1	1806
	Marovoay	Ankazomborona	20	11	466
			19	100.5	710
	Mahajanga II	Mariarano	1	441.5	4620
			18	305.6	1708
Menabe	Belo Sur Tsiribihina	Beroboka	16	26.6	1673
			12	136	521
		Tsimafana	6	96.4	2846
		Tsaraotana	3	17.7	1258
			4	57	2805
	Morondava	Befasy	11	15.4	756
			14	801.4	3565
			10	80.3	1673
			15	137.3	1001
			9	464.4	1392
			8	68.2	1374
		Bemanonga	13	356.1	1345
			2	133.2	658

## Data collection

In each study site, we first informed local officials and then selected participants. We conducted participatory mapping sessions and focus groups with the selected participants.

### Selection of participants

In each fokontany, we organized information sessions with key informants such as village chiefs, community health workers, para-veterinary assistants, and village elders to target stakeholders in the region. After discussions with these key informants, they were provided with selection criteria to select participants. Each participatory mapping and focus group discussion workshop consisted of 8 to 12 participants. Emphasis was placed on gender, given that in Madagascar, 69% of agricultural activities are managed by women^43^. The aim of selection was to reflect and mix the opinions of the participants to be sure to consider different points of view on our topic of study. First, bushpig hunters were selected as they possess valuable knowledge about bushpig population dynamics and bushpig ecology. Second, pig farmers were selected specifically for their valuable insights into interactions between domestic pigs and bushpigs, which potentially lead to the sharing of pathogens, and the occurrence of direct interactions. Third, crop farmers were included because they can provide important information about the damage caused by bushpigs and by visits by domestic pigs to their crop fields. Each participant was also able to identify the presence of hybrid pigs in the village. On average, each discussion group comprised one-third hunters, one-third pig farmers, and one-third crop farmers.

To consolidate the data collected during the workshops, we carried out a series of activities in chronological order: (*i*) presentation of the project and of the overall aims of the study, (*ii*) participatory mapping of the territory of each fokontany, (*iii*) focus group discussions using a series of semi-open questions, proportion piling and open discussions.

The sessions were conducted in Malagasy and were assisted by two local facilitators who spoke the local dialects of Sakalava Boeny and Sakalava Menabe. The participatory mapping and focus group sessions were organized in the Menabe region between August and September 2021, while in the Boeny region, they took place between October and November 2021.

### Ethics approval and consent to participate

Authorization for the study was granted by the Ministry of Agriculture and Livestock through the DSV and FOFIFA. The studies involving human participants were reviewed and approved by Ministry of Agriculture and Livestock through the DSV (Veterinary services). Ethical approval for this research study was granted by the Malagasy National Research Ethics Committee by the Ministry of Agriculture and Livestock with reference number 03–22/CENA. Local authorities were informed of the objectives and the modalities of the study prior to its initiation and were asked for permission to conduct the survey on their territory. Bushpig hunters, crop farmers and pig farmers were also informed about (i) the objectives of the study and the exclusive use of collected data for research purposes; (ii) the modalities of the studies (types of questions asked, how the interview would be conducted and its duration, how the data would be stored and kept confidential). Their participation to the interview was done on a voluntary basis, with the possibility to stop answering questions at any time. Consent of participants was expressed orally after reading accordance statement (Supplementary Information.docx Fig. 22). All research activities were performed in accordance with relevant guidelines and regulations.

### Workshop schedule

#### Participatory mapping

Participatory mapping is an effective way to visually depict important physical resources, risks, and land use patterns. It is widely recognized as a valuable tool in participatory epidemiology, as it is often easier to illustrate spatial relationships using maps than to rely solely on verbal descriptions. The mapping process can be used at the beginning of an interview to establish the spatial boundaries of the study area, as well as throughout the interview to address any spatial considerations that may arise^44^.

The method of collecting data was inspired by the methodological guide produced by the International Fund for Agricultural Development^45^. It allows local populations to spatialize local knowledge of their territory and to locate its important elements including hunting areas, agricultural practices, livestock raising practices, water courses, and access paths^46^ to assess the spatial potential of the territory^47–50^. The method comprises four stages: tracing the territory (livestock areas, hunting grounds, crop fields, etc.), taking GPS coordinates, digitalization using software, and validation.

Using a 69 × 99 cm paperboard map as a base^51^ that contained the main cartographic features of the region, including main roads, and main hydrographic network available on Open Street Map (lakes and rivers), participants were asked to delimit their territories. Tracing such areas concerned bushpig hunting trails, crop fields, water sources, protected areas, forest, pig free-range areas, areas where interactions between bushpigs and domestic pigs are likely to occur. The mapping exercise itself took approximately 90 min, including the presentation of the exercise and a break.

Based on the information mentioned by the participants (water source, crop field, etc.), each strategic location was georeferenced with a GPS, and photographed.

#### Focus group discussions

A focus group discussion is an approach used in qualitative research to gather data by engaging a specific group of individuals in a guided and in-depth conversation. This method involves the use of a moderator who guides the discussion on a particular topic or issue^52^.

Focus group discussion were organized after the participatory mapping sessions with the same participants and each session lasted an average of 90 min. Audio recording was used for analysis of interpersonal interactions.

A pilot (also termed ‘preparatory’) focus group was held before the first discussion session to be sure the questions were clear. An interview guide consisting of four sections was designed to understand the ecological factors that could play a role in the occurrence of interactions between bushpigs and domestic pigs. The first part of the session included semi-open questions on cultivation practices, characteristics of the environment surrounding each village, the occurrence and characteristics of interactions, size of the bushpig population and the pig farming system. To characterize the pig farming system, participants were asked to list the existing pig farms in their fokontany. Proportional piling according to Catley et al.^53^ was used to estimate the relative importance of each pig farming system in each fokontany investigated. Circles were drawn on a flip chart to represent each pig farming system. and the participants were asked to divide a pile of 100 beans between those different circles to represent the proportional importance of each category. The second part of the session consisted of other topics of discussion that had emerged during participatory mapping.

To characterize and evaluate the interactions, the criteria concerning the different qualitative parameters considered were explained to the participants. Direct interactions were characterized as the coexistence of bushpigs and domestic pigs in the same area at the same time, within an area equivalent to the size of a football field, which is approximately 7140 m^2^, as observed in comparable studies^54^. Indirect interactions were defined as situations where bushpigs and domestic pigs were present in the same area but at different times, as described in a study by Kukielka et al.^55^. Hotspots were defined as any area containing substrates likely to attract both species.

## Data analysis

### Participatory mapping analysis

The components that could influence interactions in each fokontany were used based on the validated map. These components included the distance from the village to the forest, the crop fields and the water source. They also included vegetation: the extent of cropland, savannah, dwellings, and forest cover. The general QGIS ‘measure’ tool^56^, which operates in a projected coordinate system, can be used to measure distances in kilometers (km) and areas in hectares (ha).

The digitized and validated mapping data were statistically analyzed using R software version 4.2.1^57^; univariate chi-square and Kruskal–Wallis tests were used to examine the data. The chi-square test was used to determine the relationship between two or more variables. The Kruskal–Wallis test was used to compare two means observed in two independent samples. The threshold for accepting the test was set at 5% (α = 0.05).

### Digitization and validation of participatory mapping

A physical copy of the map was scanned and processed to create georeferencing using ground control points through spline or first order polynomial transformations. The resulting georeferenced map was then exported to QGIS 3.10, a Coruña free Open Source software^56^ in raster format. Digital cartography made it possible to conduct ground-truthing and adding landscape features initially not included in the map. The GPS coordinates were imported into QGIS to improve the accuracy of geolocating non-georeferenced map elements. Adjusted spatialization is an important component of this activity because it is thanks to digitization that all the objects positioned on the participatory map will be found in the exact locations on the ground, with an acceptable margin of error.

Subsequent meetings were held 1 month after our first consultation with the local people. Participants reviewed and corrected the digitized paper maps showing the location of the water sources, crop fields, pigsties, pig free-range areas and interaction zones that were ranked in plenary discussions.

The results of the participatory mapping exercises based on the participants’ perceptions are presented in the form of as digitized maps in the results section. The digitized maps of neighboring villages are placed side by side or aligned vertically depending on their location. This procedure makes it possible to compare the participants' perceptions of the characteristics and use of their land with those of their neighbors.

### Typology of fokontany

Data from the semi-open-ended questions in the interview guide and from proportional piling during focus group discussion were analyzed to identify the factors that can influence interactions between domestic pigs and bushpigs in each area investigated (Supplementary Information.docx Appendix 3).

The agricultural practices, the characteristics of the environment, and the abundance of bushpigs in the vicinity of each village were assessed based on information provided by hunters, crop farmers, and pig farmers. The aim here was to assess the ecological conditions that facilitate potential interactions (Table 2).

**Table 2:** Main qualitative variables collected during focus groups with semi-open questions and used for the classification of the 20 fokontany, Madagascar.

Crops/hydrography	Location of the fokontany	Characteristics of interaction	Bushpig in the vicinity	Presence of hybrids in the village	Pig farming system
Cereals (presence/absence)	Distance between village and forest (more 500 m/less 500 m)	Observation of interactions in the last 12 months (yes/no)	Changing in the bushpig population in the last 2 years (increase/decrease/stable)	Observation of hybrids (yes/no)	Period of free ranging (rainy season/dry season/throughout year)
Fruits (presence/absence)	Distance between village and crop field (more 500 m/less 500 m)	Types of interactions observed (direct interaction/indirect interaction)	Month of bushpig visit	Heard of hybrids (yes/no)	Number of pigs in each exploitation (1–5/more 5)
Tuber (presence/absence)	Distance between village and water point (more 500 m/less 500 m)	Number of times per year there is interactions (1–5/6–10/ > 10)	Season of bushpig visit (dry season /rainy season /throughout the year)		Feeding (industrial feed/agricultural, sub-products/kitchen leftovers)
Number of fruits (categorical)		Frequency of interactions (> once more per day/ > once more per week/ > once per month)			
Fruit name (text)		Duration of interactions (< 10 min/ < 1 h/ > 1 h)			
Water sources (wells/river/river & lake)		Types of direct interactions (mating/fighting/eating together/drinking together)			
		Season of direct interactions (dry season/rainy season /throughout the year)			
		Time interval between two visits for indirect interactions			

To analyze the typology of pig farming, agreement between participant groups on the data obtained from proportional pilling with beans exercise was assessed using Kendall’s coefficient of concordance (W). Median proportion and 95% confidence interval were calculated for each pig farming system.

Multiple Correspondence Analysis (MCA)^58^ followed by hierarchical clustering on principal components (HCPC)^59^ were a method that allowed us to study the association between qualitative variables, particularly those that may influence the presence and absence of bushpigs around the fokontany, and the occurrence of interactions between domestic pigs and bushpigs (Supplementary Information.docx Appendix 1). All the variables included in the analysis were considered significant (*p* value < 0.005).

To further evaluate the direction of the potential associations between the variables recorded and the interaction between both species (the main outcome), we used the Spearman correlation between all the variables including the main outcome. To preserve as much statistical power as possible, some of the variable categories were pooled to reduce the number of categories. Then we used Fisher’s exact test to compute the estimated odds ratio of observing an interaction with each of the variables collected.

### Estimation of levels of interactions

Based on the results of the MCA and HCPC, interactions were assessed according to the qualitative estimation of their type (direct or indirect interaction), and frequency (at least once a day, at least once a week, at least once a month) and number of times per year (< 5 times a year, between 5 and 10 times a year, more than 10 times a year). The pig farming systems found in each fokontany were also included in these analyses (Supplementary Information.docx Appendix 1). Both stages of the analysis were performed in R version 4.2.1^57^ using the FactoMineR package for MCA^58^ and HCPC^59^.

### Effect size of associations

**Figure 2:**
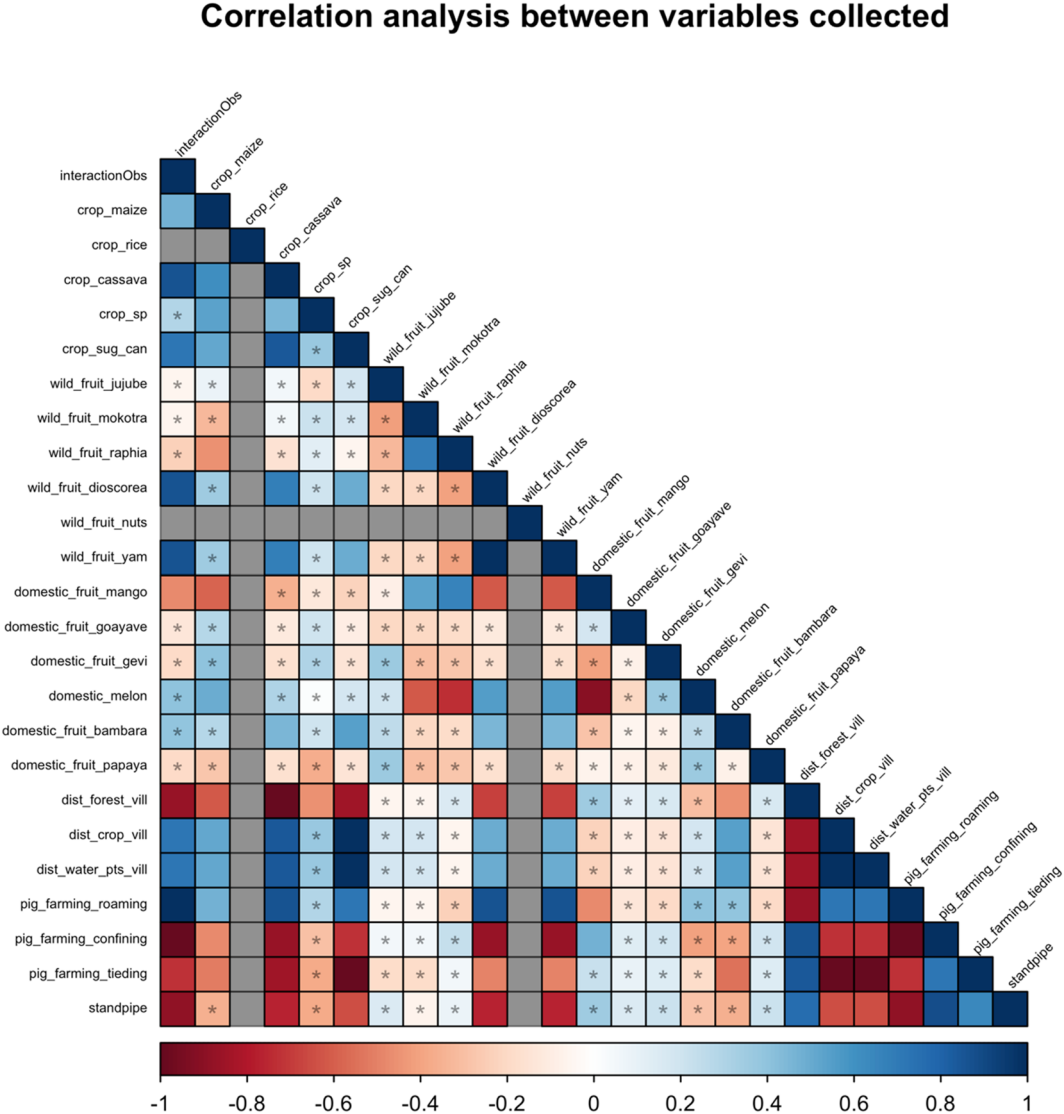
Correlation analysis between the variables collected. Colors represent the strength of the correlation, where dark red is negative correlation, dark blue positive correlation, and gray values those variables where no variability was recorded in the responses. *Denotes statistically significance.

The correlation analysis (Fig. 2) revealed a few variables with perfect correlations (both positive and negative). For instance, reports of interactions originated mainly from areas where pigs were free ranging while they were absent in areas where pigs were confined. Other variables also presented perfect correlations with variables different than the main outcome. The estimates and confidence intervals obtained from the Fisher’s exact test are presented in Supplementary Information.docx Table I. Some of the variables recorded did not had variability at all. None of the sites recorded had nuts, and all of them had rice crops.

## Results

A focus group interview and participatory mapping session were held in each fokontany (*n* = 20). The total number of participants interviewed was 241, (85 women and 156 men), including 65 hunters, 80 pig farmers, and 96 crop farmers. In each fokontany, the average number of pig farmers was 4, hunters 3, and farmers 5.

### Participatory mapping

A total of 20 maps were produced in the localities studied (Supplementary Information.docx Appendix 2). Seventy-five percent of the maps (*n* = 15) reported no interaction at all, the remaining 25% (n = 5) reported some level of interaction (direct or indirect). Four of those fokontany were located in Menabe region and one in Boeny region. Participatory mapping enabled us to obtain information on the geographical location of each fokontany, the spatialization of areas where interactions can occur, and some elements of the landscape that could influence interactions between bushpigs and domestic pigs.

Participatory mapping allowed us to characterize the landscape of the fokontany we investigated. All fokontany are located at an average distance of 1.4 km from the forest, 0.05 km from cropland, and 0.8 km from water sources. The average area of forest cover in each fokontany is 4.5 ha, the average area of cropland is 1.7 ha, while savanna occupies an average of 2.5 ha. Finally, the average area of housing and settlements is 284 ha (Table 3).

**Table 3:** Geographical features considered to influence the occurrence of interactions between bushpigs and domestic pigs.

Variables	Type of fokontany		Overall, N = 20^a^	*χ*^2^	*p* value^b^
	Without interaction N = 15^a^	With interaction N = 5^a^			
Distance village-forest (km)	1.60 (0.40, 2.40)	0.75 (0.45, 0.90)	1.40 (0.41, 1.98)	1.95	0.16
Distance village-water source (km)	0.80 (0.10, 1.45)	0.78 (0.05, 1.20)	0.79 (0.09, 1.38)	0.16	0.69
Distance village-crop fields (km)	0.20 (0.09, 0.35)	0.22 (0.05, 0.41)	0.20 (0.07, 0.40)	0.05	0.83
Crop fields area (ha)	1.479 (667, 2.531)	3.641 (1.713, 4.731)	1.676 (865, 2.874)	4.21	0.042*
Forest cover (ha)	4.247 (1.039, 6.242)	6.072 (5.246, 7.009)	4.474 (2.209, 7.110)	1.39	0.24
Savannah area (ha)	1.932 (937, 5.002)	8.034 (2.754, 9.136)	2.478 (1.277, 7.366)	3.2	0.074
Living area (ha)	242 (96, 344)	806 (551, 882)	284 (125, 536)	9.07	0.0026*

^a^Median (IQR).

^b^Kruskal–Wallis rank sum test; Kruskal–Wallis rank sum exact test.

*Significative.

In the localities investigated in Menabe region, the main crops grown were rice, corn, cassava, sweet potato, and sugarcane, all cultivated all year round. Forests were located in adjacent protected areas. The vegetation in the Boeny region consisted of a mosaic of cassava fields, rice fields, forest, and savannah.

In the five fokontany where interactions were observed, the area devoted to housing was significantly larger than in the fokontany without interactions (*p* value = 0.0026) (Table 3). There was a significant difference in the surface area of crop fields for the both types of fokontany, the cultivated area being larger in fokontany with reported interactions (*p* value  = 0.042).

When we compared distances between the fokontany and the nearest forest, the nearest water source, and the nearest crop fields, we found no significant differences between fokontany with and without reported interactions. Despite the surface of forest cover was different in both fokontany (with and without interaction), the difference was not statistically significant. Neither was there any significant difference in the extent of the savannah when we did the same comparison.

Bushpigs were systematically hunted in fokontany that included forest within a median radius of 0.75 km (*n* = 5) and bushpigs were more commonly reported around the village. In other villages, locations reported as frequented by bushpigs included crop fields, water sources, but also savannah. In places where the distance between the village and the edge of the forest was bigger (on average above 15 km), bushpigs rarely ventured close the village.

**Figure 3:**
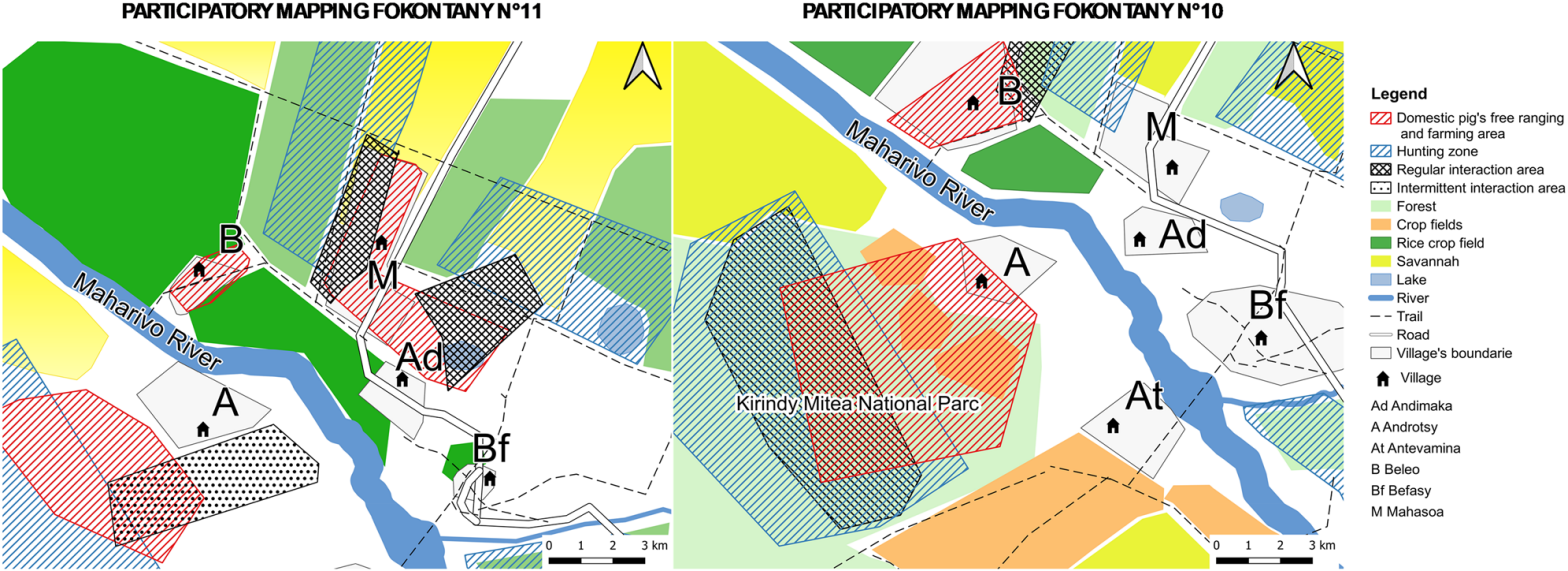
Example of overlaying two maps with high interactions. The base map was created with QGIS software version 3.10 a free opensource software http://qgis.org/, using shapefiles containing the main roads and water networks available on https://openstreetmap.org/. The map was exported from QGIS in Pdf format and printed on 69 × 99 cm paper for using in the fieldwork and to be manually annotated during participatory mapping. Using the basemap, participants characterised their territories, a physical copy of the map was scanned and processed to create georeferencing using ground control points through spline or first-order polynomial transformations. The resulting georeferenced map containing the features plotted by the participants was then exported and processed in QGIS. Participants reviewed and corrected the scanned paper maps.

**Figure 4:**
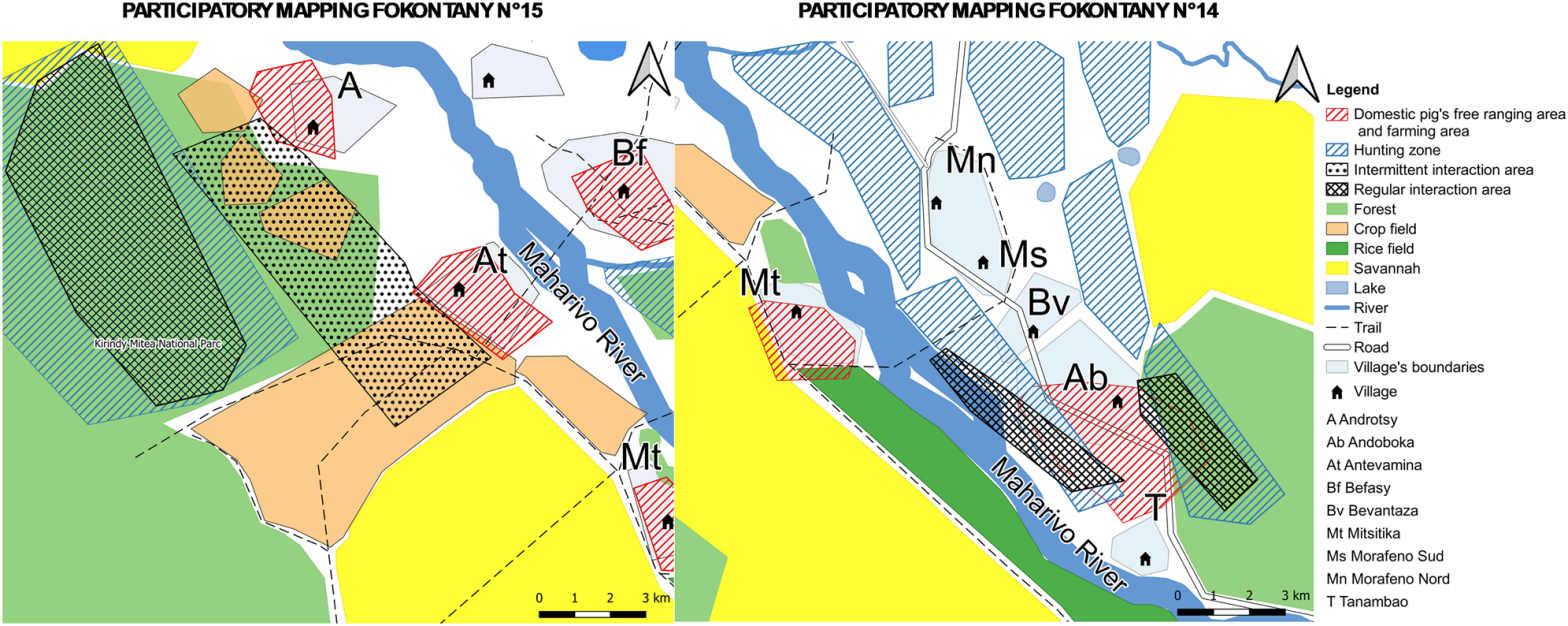
Example of overlaying two maps with high and intermediate interactions. The base map was created with QGIS software version 3.10, a free opensource software http://qgis.org/, using shapefiles containing the main roads and water networks available on https://openstreetmap.org/. The map was exported from QGIS in Pdf format and printed on 69 × 99 cm paper for using in the fieldwork and to be manually annotated during participatory mapping. Using the basemap, participants characterised their territories, a physical copy of the map was scanned and processed to create georeferencing using ground control points through spline or first-order polynomial transformations. The resulting georeferenced map containing the features plotted by the participants was then exported and processed in QGIS. Participants reviewed and corrected the scanned paper maps.

Pig free ranging areas and pig paddocks were mainly located within the village or crop fields within an average radius of 1.5 km from the village. In the villages where interactions were reported, they occurred mainly in the savannah and near water sources such as rivers, ponds, or lakes. In other localities, interactions were reported near the village, in crop fields, but also near the closest forests (Figs. 3, 4).

**Figure 5:**
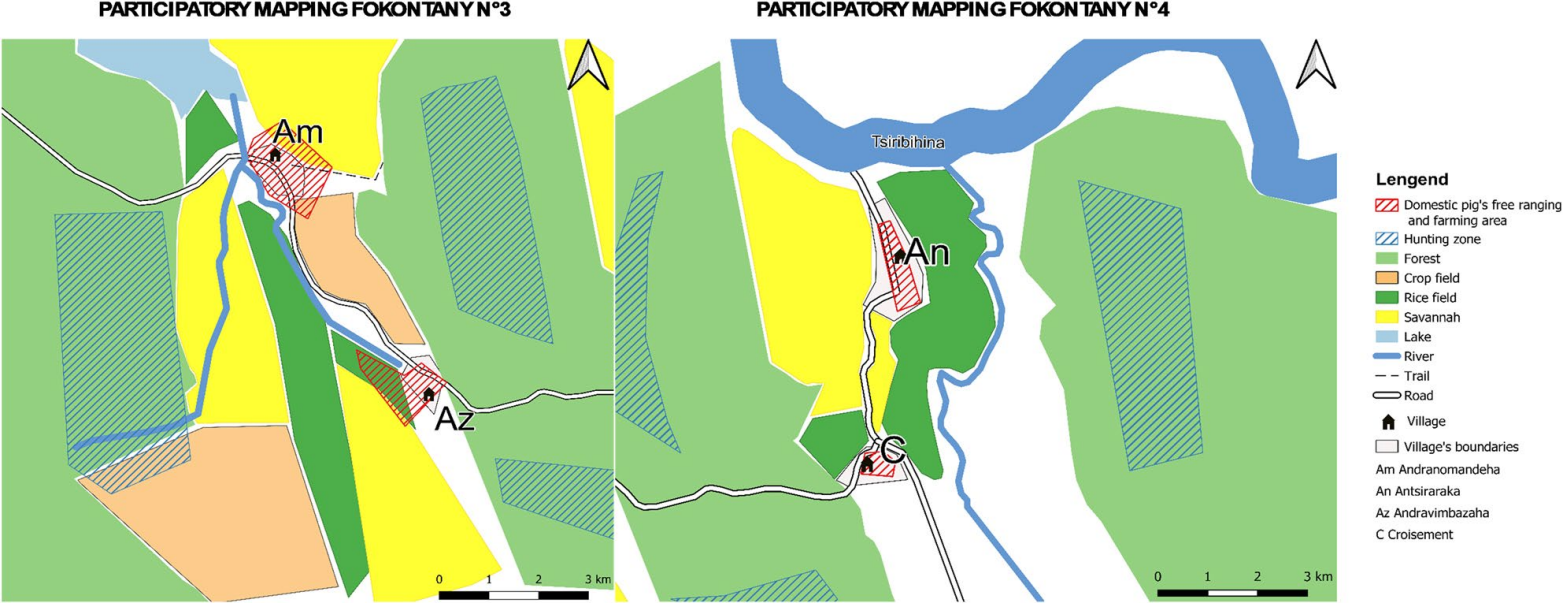
Example of overlaying two maps without interactions. The base map was created with QGIS software version 3.10, a free opensource software http://qgis.org/, using shapefiles containing the main roads and water networks available on https://openstreetmap.org/. The map was exported from QGIS in Pdf format and printed on 69 × 99 cm paper for using in the fieldwork and to be manually annotated during participatory mapping. Using the basemap, participants characterised their territories, a physical copy of the map was scanned and processed to create georeferencing using ground control points through spline or first-order polynomial transformations. The resulting georeferenced map containing the features plotted by the participants was then exported and processed in QGIS. Participants reviewed and corrected the scanned paper maps.

The interactions were characterized by an overlap between the places visited by bushpigs and the places where domestic pigs roamed freely. These areas are usually located near a forest, a water source or a crop field but also in the savannah. In the non-interaction sites (Fig. 5), an average of 2 km was observed between the sites frequented by bushpigs and the sites of pig farms and pigs. In general, the two types of sites were separated by a stream or a river and sometimes also by a road.

### Focus group interviews

#### Typology of pig farming practices

Estimated through proportional piling, the relative variations in confined pig farming, free-range pig farming and systems in which pigs were tied to stakes was 18.8, 21.35, and 60.35%, respectively (Table 4). There was good agreement between the participants in 20 fokontany (W = 0.86, *p* < 0.001).

**Table 4:** Summarized median scores (95% CI) indicating the relative importance of pig farming system as determined through proportional pilling exercise in the fokontany investigated, Madagascar.

Fokontany (n = 20)	Farming system^a^		
	Confined rearing all day	Free ranging all day	Raising pigs tied to stakes
1	23 (15–31)	1 (0–4)	76 (65–84)
2	25 (19–33)	0 (0–4)	75 (55–75)
3	24 (20–30)	5 (2–8)	71 (65–78)
4	25 (21–30)	0 (0–5)	75 (61–83)
5	29 (23–41)	0 (0–6)	81 (68–83)
6	26 (19–33)	3 (0–6)	71 (60–75)
7	25 (20–29)	1 (0–3)	74 (70–77)
8	24 (20–29)	5 (2–7)	71 (68–73)
9	25 (22–30)	0 (0–6)	75 (70–78)
10	0 (0–5)	51 (40–56)	49 (39–55)
11	0 (0–3)	100 (85–100)	0 (0–2)
12	23 (12–27)	7 (3–9)	70 (55–75)
13	29 (20–31)	3 (1–9)	68 (65–71)
14	1 (0–4)	99 (76–100)	1 (0–4)
15	1 (0–25)	50 (40–53)	50 (48–56)
16	25 (22–33)	0 (0–4)	75 (69–78)
17	0 (0–3)	98 (87–100)	2 (0–4)
18	25 (19–30)	3 (0–5)	72 (55–78)
19	23 (25–31)	1 (0–4)	76 (66–78)
20	25 (14–30)	0 (0–5)	75 (65–87)
Overall mean (%)	18.8	21.35	60.35

There is good agreement between informant groups (W = 0.86).

n = number of fokontany where the exercise was conducted.

^a^The figures represent the median scores, and 95% confidence interval are showed in parentheses.

#### Typology of fokontany

**Figure 6:**
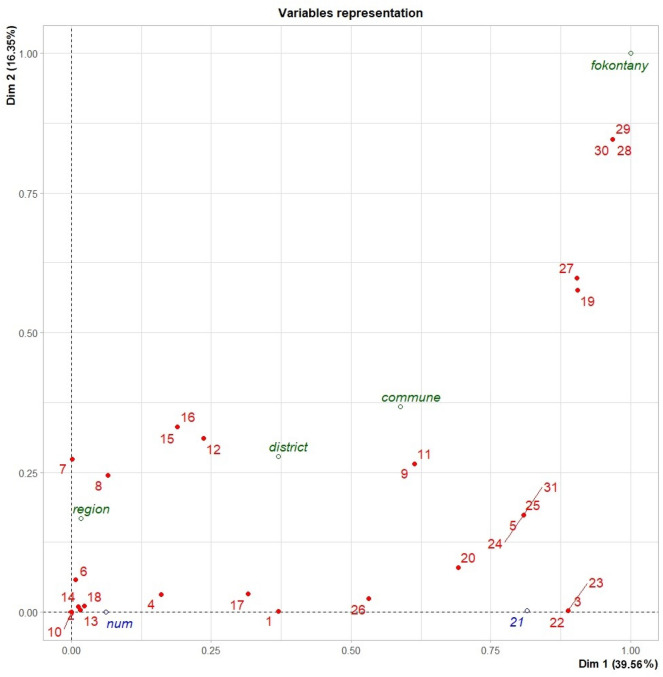
Dimension map 1 and 2 of each analyzed variable (in red the variables of the analysis, in green the supplementary variables and in blue supplementary numerical variables). ^1^Maize, ^2^Rice, ^3^Cassava, ^4^Sweet potatoes, ^5^Sugar cane, ^6^Jujube, ^7^Mokotro, ^8^Raphia, ^9^Dioscorea, ^10^Nuts, ^11^Yam, ^12^Mango, ^13^Guava, ^14^Gevi, ^15^Melon, ^16^Watermelon, ^17^Bambara, ^18^Papaya, ^19^Water sources, ^20^Bushpig population, ^21^Number of months of bushpig presence around the village, ^22^Bushpig season visit around the village, ^23^Distance between forest and village, ^24^Distance between crop and village, ^25^Distance between water source and village, ^26^Observation of hybrids in the village, ^27^Observation of interactions in the last 12 months, ^28^Frequency of interactions, ^29^Number of interactions per year, ^30^Pig farming system, ^31^Pig population.

After integrating data on agricultural practices, environmental characteristics, bushpig populations, and the pig farming system into the MCA analysis, 5 dimensions were selected for HCPC analysis that explained 81% of variance in the data (Supplementary Information.docx Appendix 1). Each of these dimensions alone explained more than 5.5% of total variance. The variables with the most impact on these dimensions were distance between the forest and the village, the seasons of bushpig presence and observation (Fig. 6). The HCPC analysis then enabled the distribution of the villages into 3 clusters (Supplementary Information.docx Fig. 6). All the fokontany and the different values of the initial variables are represented on the same plane defined by the 5 dimensions retained (Supplementary Information.docx Appendix 1).

The most frequently represented (75%) type of fokontany was cluster 1, corresponding to fokontany located at a distance of more than 500 m from the forest, less than 500 m from crop fields and water points consisting exclusively of wells (Table 5). In this cluster, bushpig visits occurred in the dry season, on average over a period of one to 4 months per year. Most of these fokontany reported a decrease in the bushpig population in the last 2 years, and hybrid pigs were reported in only 14% of them. These areas lacked wild fruits or sugar cane, with sweet potato being the primary tuber grown. The majority of pigs in this cluster (about 75%) were confined year-round, while the rest are tethered during the rainy season and fed a specific diet, typically numbering between one to five pigs per farm.

**Table 5:** Simplified description of clusters according to crops, water point presence of bushpigs.

Cluster	Wild fruit	Number of wild fruits in each village	Crop: tuber	Crop: sugarcane	Number of crops in each village	Distance village- water points	Water points	Distance village-forest	Distance village-crop fields	Number of months of bushpig presence around the village	Hybrid observation	Population of bushpigs	Observation of interactions in the last 12 months	Types of interactions observed	Frequency of interactions	Number of times per year	Pig farming system
1	Absent	0	Sweet potato (47%)	Absent	0_1	Less 500 m	Public wells	More 500 m	Less 500 m	< 1–4 months	No (87%)	Decrease (73%)	No	None	NA	NA	75% pigs confined 25% pigs tied
											Yes (13%)	Stable (20%)					
												Increase (17%)					
2	Jujube	1	Yam (50%) Cassava (50%) Sweet potato (50%) Taro (100%)	Absent	< 2–5	Less 500 m	River	Less 500 m (50%)	Less 500 m	< 4–7 months	Yes (100%)	Increase (100%)	Yes	Indirect interaction (100%)	At least once per month (100%)	06_10	50% pigs roamed 50% pigs tied
								More 500 m (50%)									
3	Dioscorea Mokotra Raffia Jujube	> 2	Yam (100%) Cassava (100%) Sweet potato (100%) Taro (100%)	Present	> 5	More 500 m	River and lake	Less 500 m	More 500 m	> 7 months	Yes (100%)	Increase (100%)	Yes	Indirect interaction (80%)	More than once per week (80%)	> 10	All pigs roamed
														Direct interaction (20%)	More than once per week (20%)		

Cluster 3, comprising 15% of the fokontany, included fokontany located less than 500 m from the forest and crop fields growing maize, yam, sugar cane, and cassava. Water sources were situated over 500 m away and mainly consisted of a lake and/or a river (Table 5). Wild fruits like jujube, raffia fruit, and mokotra grew nearby, along with various tubers such as yam, cassava, sweet potato, and taro. In these fokontany, bushpig populations had increased over the past 2 years, and hybrid pigs were reported. Bushpig visits occurred year-round for an average of over 7 months. Interactions between bushpigs and domestic pigs, including mating attempts and fights, were common, happening more than ten times annually, mostly in crop fields at night. Pigs in this cluster roamed freely throughout the year and were occasionally fed agricultural by-products. The number of pigs on each farm ranged from 5 to 10.

Finally, cluster 2 came in last (10%) and only comprised two fokontany, both located in the Menabe region. These fokontany resembled cluster 3 in terms of distance separating villages from water points, crop fields and the forest (Table 5). The water sources used by the villagers in this cluster were exclusively rivers. In these fokontany, only one kind of wild fruit (jujube) was present. Bushpigs visited the villages on average 4–7 months per year. Indirect interactions were the only type reported by villages in this cluster. Interactions were detected 6 to 10 times per year. However, hybrids were observed in these fokontany. Over half of the pigs in this cluster roamed freely year-round, while the remainder were tethered during the rainy season. The animals were fed mainly with agricultural by-products and kitchen waste. The number of pigs per farm ranged from 1 to 5.

#### Levels of interactions based on participants knowledge

The results of participatory mapping allowed us to spatialize the likely locations of interactions between bushpigs and domestic pigs. Analysis of the participants’ responses to the semi-open questions asked during the focus groups allowed us to identify three types of zones based on the qualitative level of interaction: zones with no interactions, zones with an intermediate level of interactions and zones with a high level of interactions. Based on the MCA and HCPC classifications, three groups emerged depending on the variables that determine the interactions: (i) *high level of interactions* was defined as the occurrence of an interaction and an indirect interaction more than 10 times a year. Indirect interactions occurred once a week; (ii) *intermediate level of interactions*, the presence of indirect interaction between the two species occurred at least once a month, with an average frequency of 6 to 10 times a year; (iii) *no interaction:* neither direct nor indirect interactions occurred. The zones without interactions are in fokontany encompassing Cluster 1. The zones with intermediate interactions are in fokontany encompassing in Cluster 2, all in Menabe region. Finally, areas with a high level of interactions are in Cluster 3 in fokontany located in both Menabe and Boeny regions.

## Discussion

This is the first study to attempt to characterize the interactions between domestic pigs and bushpigs using a participatory approach in a wildlife-domestic interface in Madagascar. Our results are important to considerably advance our understanding of the behavior of bushpigs in proximity of human settlements which is poorly documented in the literature. Indeed, the two species of *Suidae* have been suspected to interact in Madagascar^60^ and in some parts of the African continent^61,62^ and potentially share a certain number of pathogens.

Our results confirm that participatory epidemiological approaches are both useful and appropriate for characterizing interactions between these two species. Participatory mapping is a process of discussion with local people to spatialize interactions and understand the relationships between the structure of each fokontany and the landscape elements that may influence interactions between bushpigs and domestic pigs. The combination of the two approaches is one originality of our study that allowed us to increase and deepen our knowledge of local communities. This approach has also been reported elsewhere to be a useful and cost-effective way to collect qualitative and quantitative information on events that are otherwise difficult to observe. These approaches allowed us to spatialize potential pig free ranging areas, as well as the preferred locations of bushpigs in fields. The production of the 20 maps enabled us to discuss with stakeholders and as a result, to identify spatially and consensually, hotspots for future interaction studies. Our maps may not have captured changes in activities with changes in the season. For example, free-range areas used by pigs are not permanent and their activity may change over the course of the year. Differences are also possible in their choice of crop, which are also selected according to the season.

Furthermore, our study emphasizes that local knowledge can be used to implement collective strategies for managing wildlife-domestic animal health risks. This study not only increases our knowledge of the interactions between wildlife and domestic animals in potential interface areas, it also reveals that pig farmers have precise knowledge of what is happening with their animals in and around their farms. The gender ratio was not totally respected, despite our efforts to encourage the participation of women during courtesy visits to each fokontany. However, we think without our efforts in that respect women participation would have been even lower, as Malagasy society tends to exclude women and favor men in community meetings^63^.

Our study provides a good level of basic information at low resolution on the geographic and socio-ecological landscape that could be the scene of interactions between domestic pigs and bushpigs in selected rural areas of Madagascar. The absence of interaction between both species was reported in 3/4 of the fokontany studied. This is not surprising considering that a large part of the fokontany interviewed (a total of 85%), pigs were kept under free ranging conditions. However, it should be also be taken into consideration that bushpigs being mainly nocturnal^64^, interactions occur at night and hence are difficult to observe. The low level of interactions observed (only 25% of the farmers in the locations we studied reported some level of interspecific interactions) partially confirms this hypothesis. Our results also indicate that the majority of interactions reported are indirect and are triggered by access to sites that attract both species, such as trophic resources or water, but that may be visited at different times.

The relatively small sample size resulted into extreme estimates and *p* values, nevertheless the direction of the associations observed supports the current known drivers of interactions. Based on our findings, the degree of interactions could have been influenced by the number of pigs that roamed freely in each fokontany. Free ranging pigs can cover considerable distances (between 1 and 4 km) in search of food and water, if these are not provided in sufficient quantities by their owners^23,65^. Indeed, the probability of encounters with bushpigs probably increases when pigs have the opportunity to search for feed in crop fields and forests. These observations are consistent with those made by Okoth et al. in rural Kenya, who described similar interactions between free-ranging pigs and bushpigs, especially at night^66^. In our case, the proximity of protected areas in both study sites may have contributed to the abundance of bushpigs, facilitating interactions with free ranging domestic pigs in the vicinity^20,55,66,67^ The dependence on forest for cover is common to many other wild Suidae including wild boar in Europe^68,69^, giant forest-hog in West and Central Africa^70^, and in East and Southern Africa^71^. Similarly, there were no reports of fruit trees growing around villages without interactions, whereas their presence seemed to play an important role in attracting both species. Fruit tree species such as jujube, raffia and mokotro, which are prized not only by bushpigs^37,55,72^ but also by free ranging pigs^65,73,74^.

Another trophic resource that could have facilitated interspecific interactions is the availability of agricultural crops. In the fokontany in which high levels of interactions occurred, several types of crops were grown in the vicinity of the village, including maize, cassava, tubers, and sugarcane. In areas where intermediate interactions occurred, fewer trophic resources were available, while in areas with no interactions, only sweet potatoes were planted close to the village. Bushpigs are known to be major crop raiders throughout their distribution area^55,75,76^ and our results confirm this pattern. Crop fields also play a role as interaction hotspots when they coincide with free-ranging pigs, as already observed in Uganda^67^. Our results also indicate that the level of interactions varies with the availability and proximity of water. In the first case, water resources including rivers, and lakes are critical for any pig species and likely to represent attraction hotspots. Similar patterns have been observed in the case of direct or indirect interactions between free ranging domestic pigs and wild boars (*Sus scrofa*) in Spain^77,78^, particularly in the dry season. A very similar finding was reported in Uganda, in that case involving bushpigs and domestic pigs^55^.

In our study, direct interactions reported in 3 of the fokontany were related to sexual attraction between male bushpigs and domestic sows in heat or fights between male bushpigs and domestic boars (Table 5). This hypothesis is further supported by reports of hybridization in 30% the fokontany studied. These reports often referred to female sows being mated by male bushpigs when left unattended for a several weeks while farmers were busy harvesting the crops earlier in dry season. Sexual interactions and hybridization between wild boars (*Sus scrofa*) and domestic pigs are widely documented in Europe^79–81^, although in this case, they surprisingly involve two different suid species. In addition, anecdotical reports of *Potamochoerus sp.* cross breeding with domestic pigs are repeatedly mentioned in the literature in several parts of sub-Saharan Africa^62,82,83^. Moreover, a recent paper exploring the genetic diversity of Kenyan domestic pigs reported evidence of genetic introgression of bushpig genes in several individual domestic pigs sampled^84^. However, this hybridization phenomenon has never been to date demonstrated scientifically with robust genetic tools.

Sharing habitat and resources between wild suids and domestic pigs can lead to the transmission of diseases^7,80,85,86^. Bushpigs and domestic pigs, being in the same taxonomic group, share various diseases, including ASF^12,66,87,88^. Other zoonotic diseases of public health concern can also be transmitted through handling and consumption of carcasses of infected bushpigs. These diseases include bovine tuberculosis^89^, trichinellosis^90^ or helminthiasis^36^. In the context of our study, environmental transmission of these resistant pathogens is likely. Serological evidence of exposure of bushpig to toxoplasmosis was also recently detected in Madagascar [Rakotoarivony, personal observation].

A potential bias in our results is that study areas were chosen using non-random, purposive sampling and identified 'information-rich cases' based on the recommendations of local stakeholders. In this context, possible overestimation of the level of interaction would have been expected but was not confirmed by our results, which only showed a small proportion of indirect (10%) and direct interactions (15%). This confirms the hypothesis that such situations was relatively exceptional unless pigs were allowed to range completely freely^55^ combined with abundant bushpig populations. From that perspective, the fact of deliberately choosing study areas that facilitated these interactions proved useful to collect local knowledge concerning those rare events. Another potential bias in our results is the influence of the researchers’ opinions on the perceptions and on the views expressed by the participants. In this case, it is possible that the case of interspecific interactions was reported by local populations to please researcher objectives, but in fact did not happen. However, that hypothesis is with agreement between reports of sightings by different fokontany to some extent confirmed the veracity of the reports. Indeed, some of the digitized maps of neighboring villages were placed side by side or aligned vertically, depending on their location, allowing the participants to confirm their observations by spatial triangulation. This was for instance the case between fokontany n°14 and n°15 in Menabe region (Fig. 3).

This research has documented the occurrence of a certain level of interactions and its potential health implications. This information can be used to target awareness campaigns to highlight the health implications of improving biosecurity measures in small scale animal farming systems and reduce the transmission of infectious and parasitic diseases at the wildlife/livestock/human interface^91,92^. This approach could also be replicated or adapted to similar situations in other parts of the world in where wild and domestic species interact such as the case of domestic and wild bovids and the shared transmission of foot and mouth disease^93,94^, or the case of domestic wild birds and the shared circulation of Avian Influenza^95,96^.

## Conclusion

Gathering local knowledge using a combination of participatory approaches such as participatory mapping and focus groups proved useful to describe the socio-ecological context in which bushpigs and domestic pigs could potentially interact. The combination of tools enabled us to capture exceptional information on the occurrence of events, which are otherwise rarely observed and described in the literature. In addition, the reported observations made biological sense and were consistent with previous reports on the behavior and ecology of wild suids. Fruit trees, crops, and water sources were identified as attracting both species and hence, potentially acting as hotspots of interactions in places where domestic pigs are allowed to range freely. Reports of sexually driven direct interactions between the two species were confirmed by reports of presence of suspected hybrids in seven locations. Further research using more sophisticated tools such as camera traps and genetic analysis of suspected hybrids with powerful genomic tools should be able to confirm or infirm some of our observations data on. In the meantime, the local knowledge gathered in our study clearly demonstrates that in rural Madagascar, both species are attracted by several available resources (crops, forest, fruit trees, and water sources) that allow them to interact, and potentially exchange different infectious pathogens. The implications of our results are highly relevant to make decisions and inform policies or strategies on management and of disease circulating at the wildlife/livestock/human interface.

### Supplementary Information

Supplementary Information.

## Data Availability

The data that support the findings of this study are available on reasonable request from the corresponding author, RR. None of the data are publicly available because they contain information that could compromise the privacy of the research participants.
