# Supplementary material for: Assessment of domestic pig–bushpig (Potamochoerus larvatus) interactions through local knowledge in rural areas of Madagascar
Source: Sci Rep. 2024 Jul 15;14:16310. doi: 10.1038/s41598-024-67208-1 (PMC11250805; doi:10.1038/s41598-024-67208-1)
Supplement: Supplementary file 1 — Supplementary Information. [file 41598_2024_67208_MOESM1_ESM.pdf]

SUPPLEMENTARY INFORMATION

Assessment of domestic pig - bushpig (Potamochoerus larvatus) interactions through local knowledge in rural areas of Madagascar

Rianja Rakotoarivony[1,2*], Daouda Kassie [1,3], Alpha Andriamahefa [2], Diana Andria-Mananjara [2], Mihaja Rakotoarinoro [2], Herilanto Ramaroson [2], Modestine Raliniaina [2], Miatrana Rasamoelina [2], Jose Pablo Gomez-Vazquez [4] and Ferran Jori [1,5]

[1] Joint Research Unit-Animal-Health-Territories-Risks-Ecosystems (UMR ASTRE), CIRAD, Campus International de Baillarguet, Montpellier, France, [2] National Centre for Applied Research in Rural Development- Department of Zootechnical Veterinary and Fish Farming Research (FOFIFA-DRZVP), Antananarivo, Madagascar, [3] Institut Pasteur de Madagascar, Antananarivo, Madagascar, [4] Center for Animal Disease Modeling and Surveillance, Department of Medicine and Epidemiology, School of Veterinary Medicine, University of California Davis, Davis, USA, [5] Department of Zoology and Entomology, University of Pretoria, Pretoria, South Africa

Appendix 1: MCA and HCPC approaches

MCA is generally used to identify the underlying structure of the data. It allows both to identify associations between parameters and to determine groups of areas (clusters) with similar profiles in their practices and characteristics [1]. This approach is based on the fact that each area is represented according to its characteristics in a Euclidean plane defined by specific dimensions. These dimensions, also called components or projections, are defined in such a way as to best capture the variance of the variables. They are therefore linear combinations of the variables under study representing different percentages of the variance (characterized by the eigen values). The MCA can therefore be interpreted as an orthogonal decomposition of the variance of a database. In our project, the MCA is a preliminary step to the realization of clusters of qualitative data. In a second step, a hierarchical classification using Ward's method is performed on the results of the MCA to identify groups of areas sharing the same characteristics [2].

From the preliminary bibliography, we selected 38 factors (Table II in manuscript) that were suspected to play a role in the interactions and to allow a good description of the localities. Six other factors are included in the analysis as additional variables because of a lack of interest for the analysis (e.g., region, district, municipality, fokontany) or because the variable is a redundancy of responses (e.g., list of fruits, number of fruits present). These additional variables are not involved in determining the dimensions and thus the analysis but can be projected on these components to see how they relate to the other variables and the groups of localities.

Based on the results of the MCA, we decided to keep the first 5 dimensions, explaining a sufficient percentage of the variance for further analysis (Figure 1). The determination of the number of groups retained for further HCPC analysis was then made based on the statistical results and visual inspection of the overall appearance of the hierarchical tree [3].

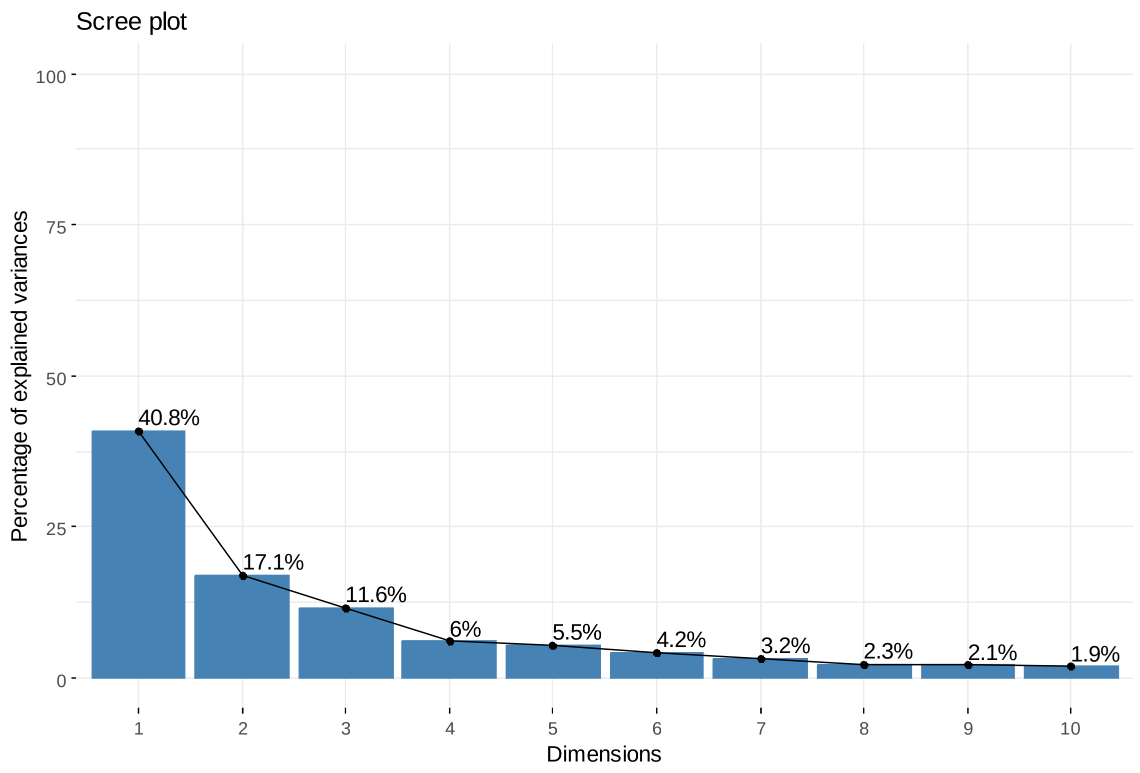

Figure 1: Results of the multiple correspondence analysis: percentage of variance explained by each dimension

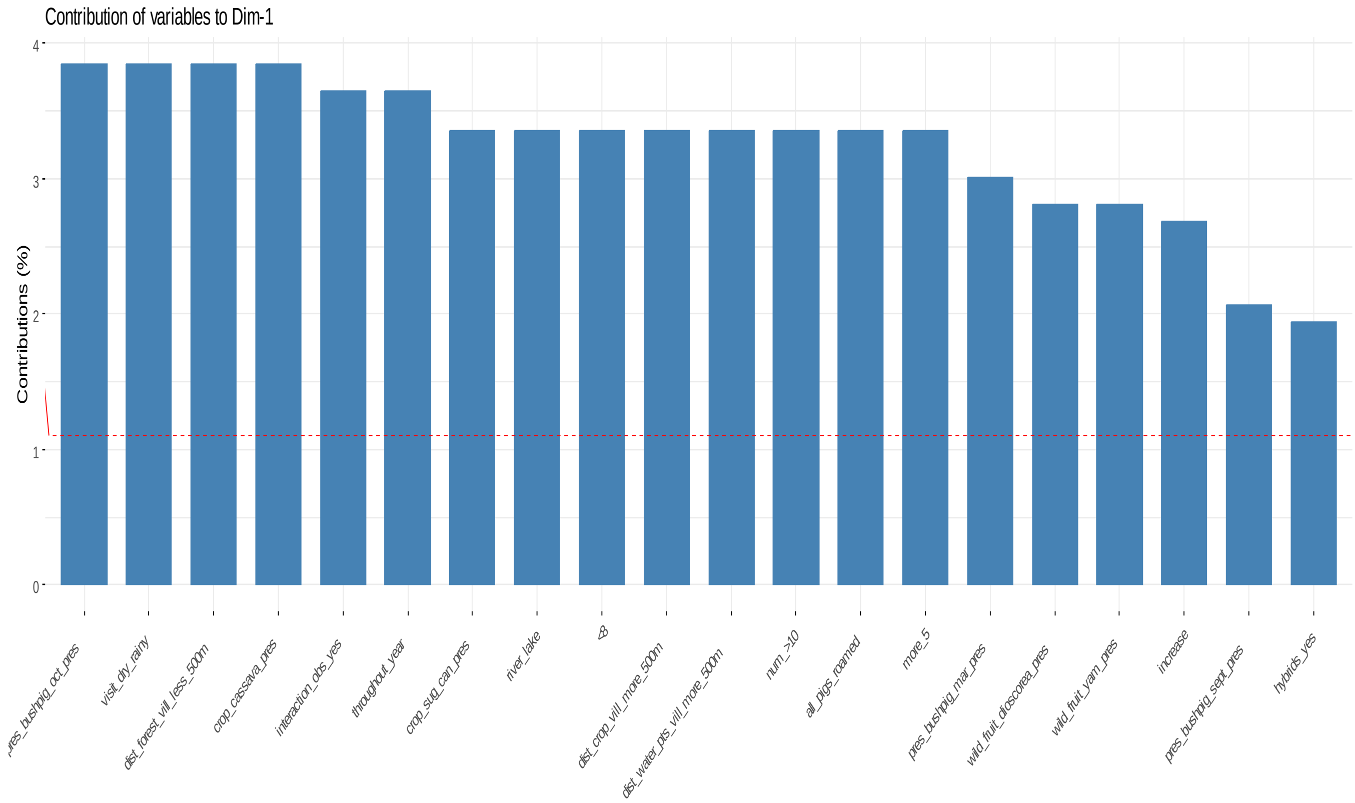

Figure 2: Results of the MCA analysis: contribution of the variables to dimension 1

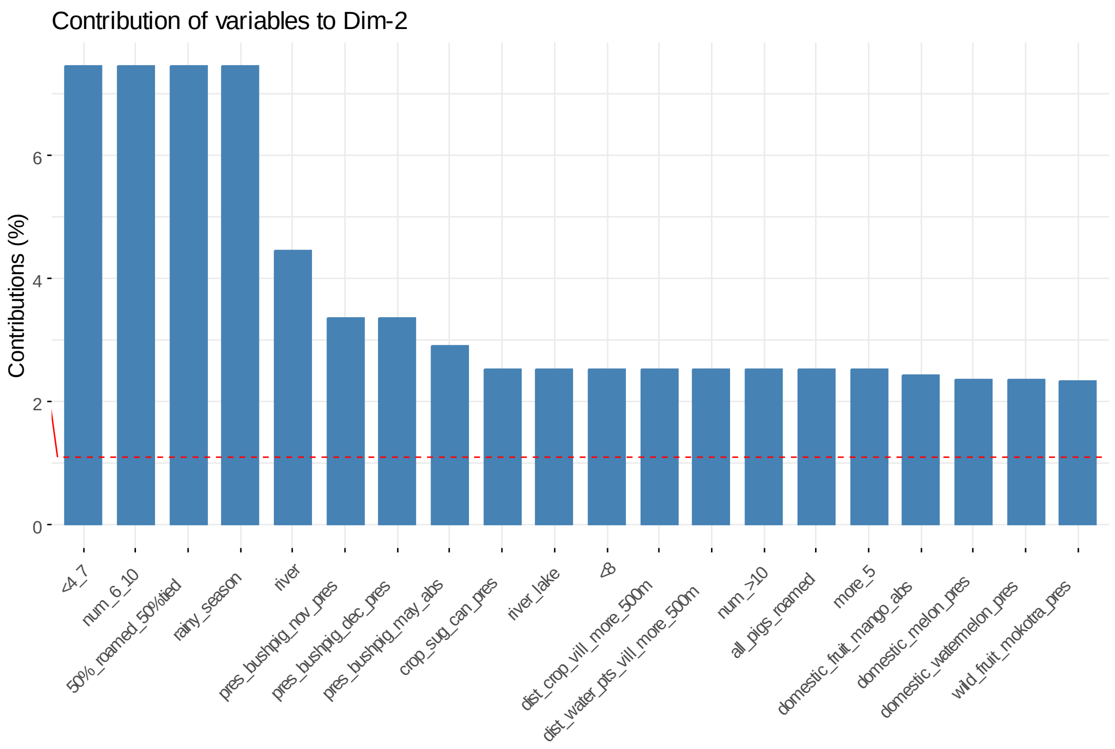

Figure 3: Results of the MCA analysis: contribution of the variables to dimension 2

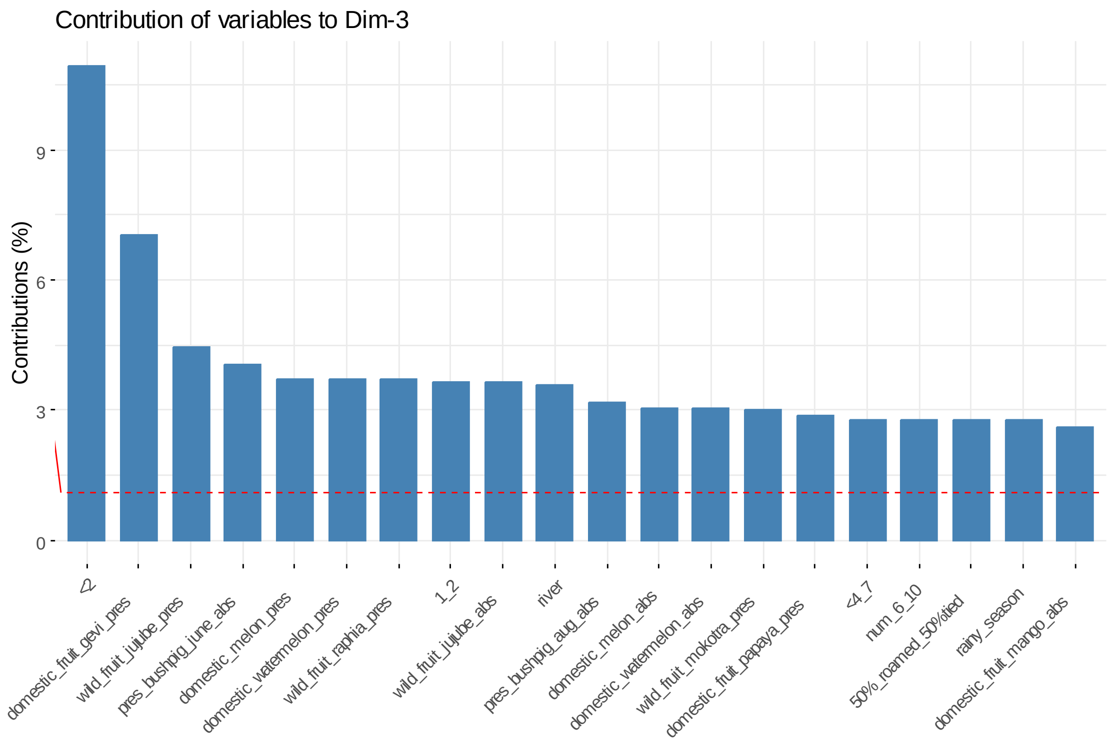

Figure 4: Results of the MCA analysis: contribution of the dimension 3

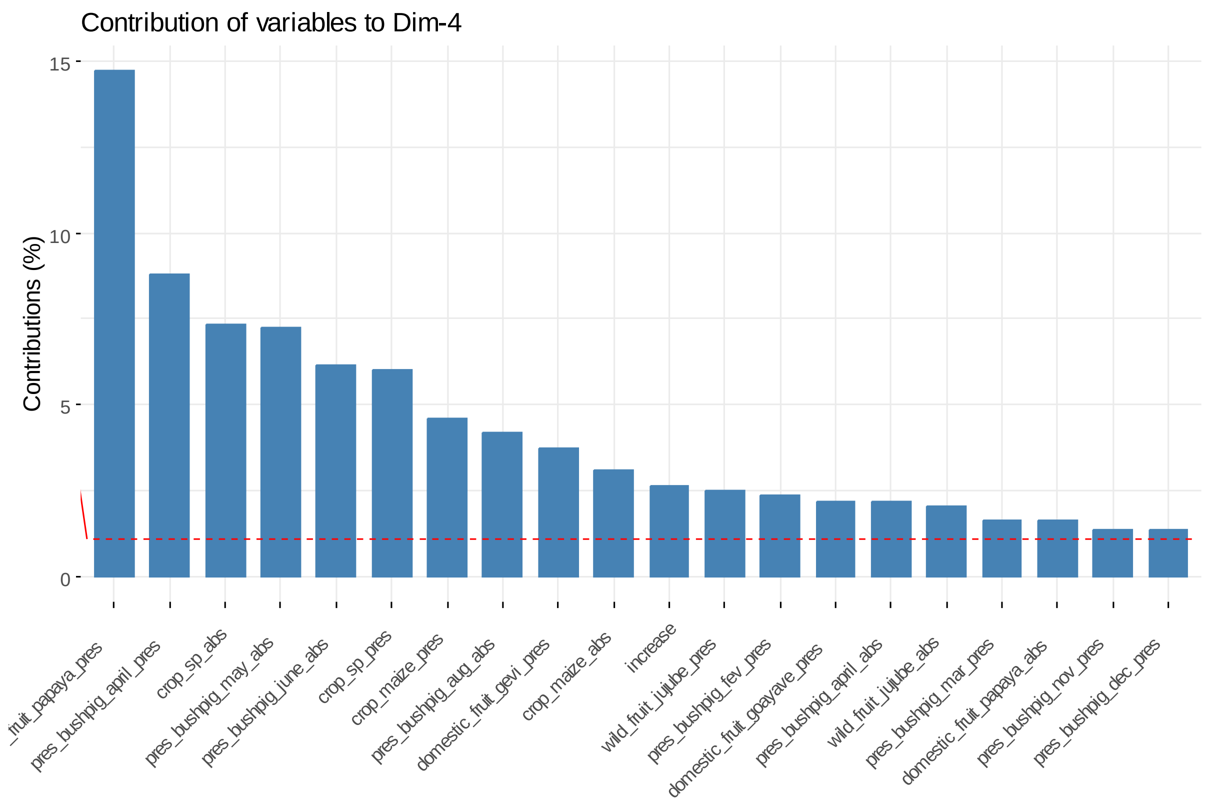

Figure 5: Results of the MCA analysis: contribution of the dimension 4

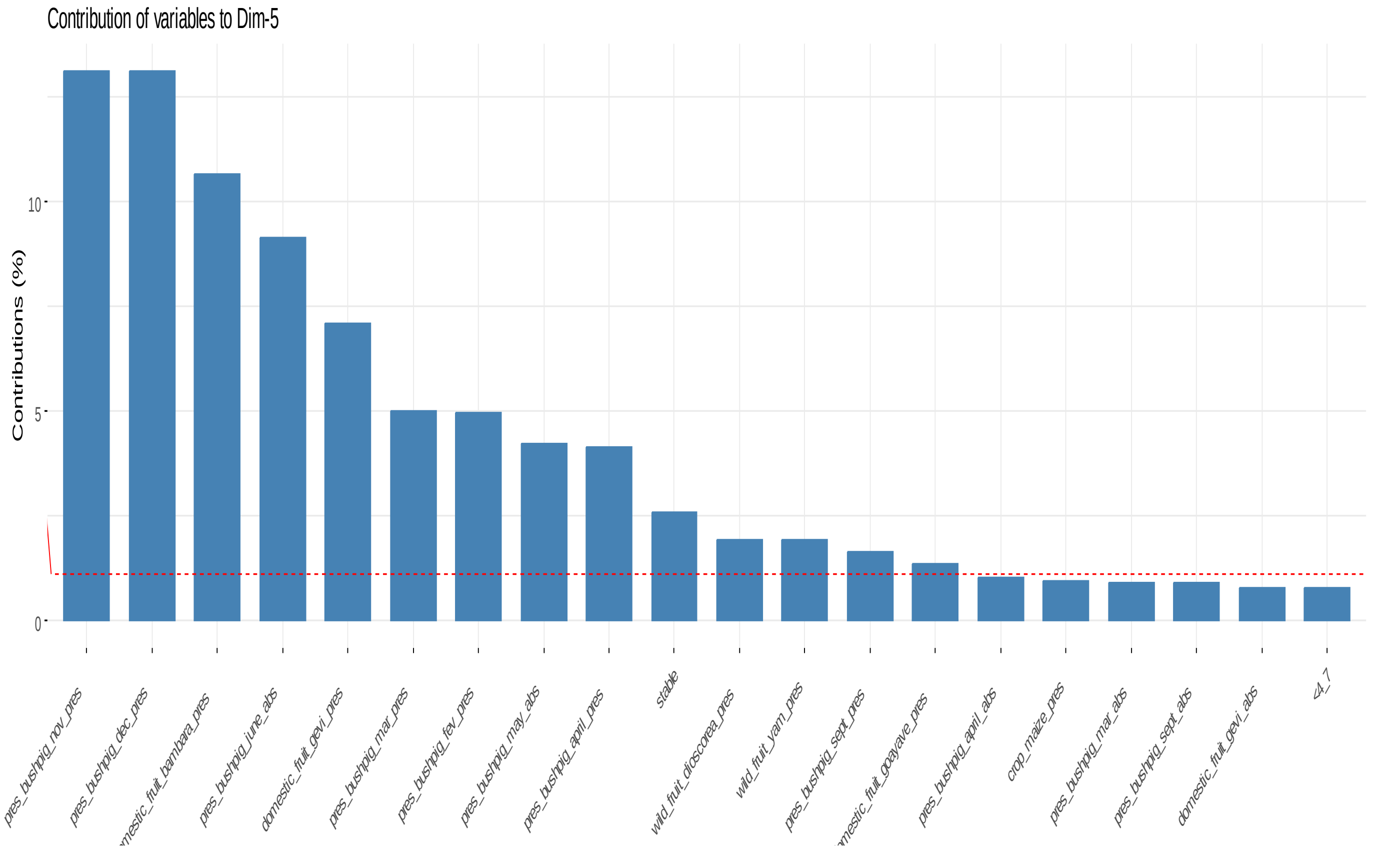

Figure 6: Results of the MCA analysis: contribution of the dimension 5

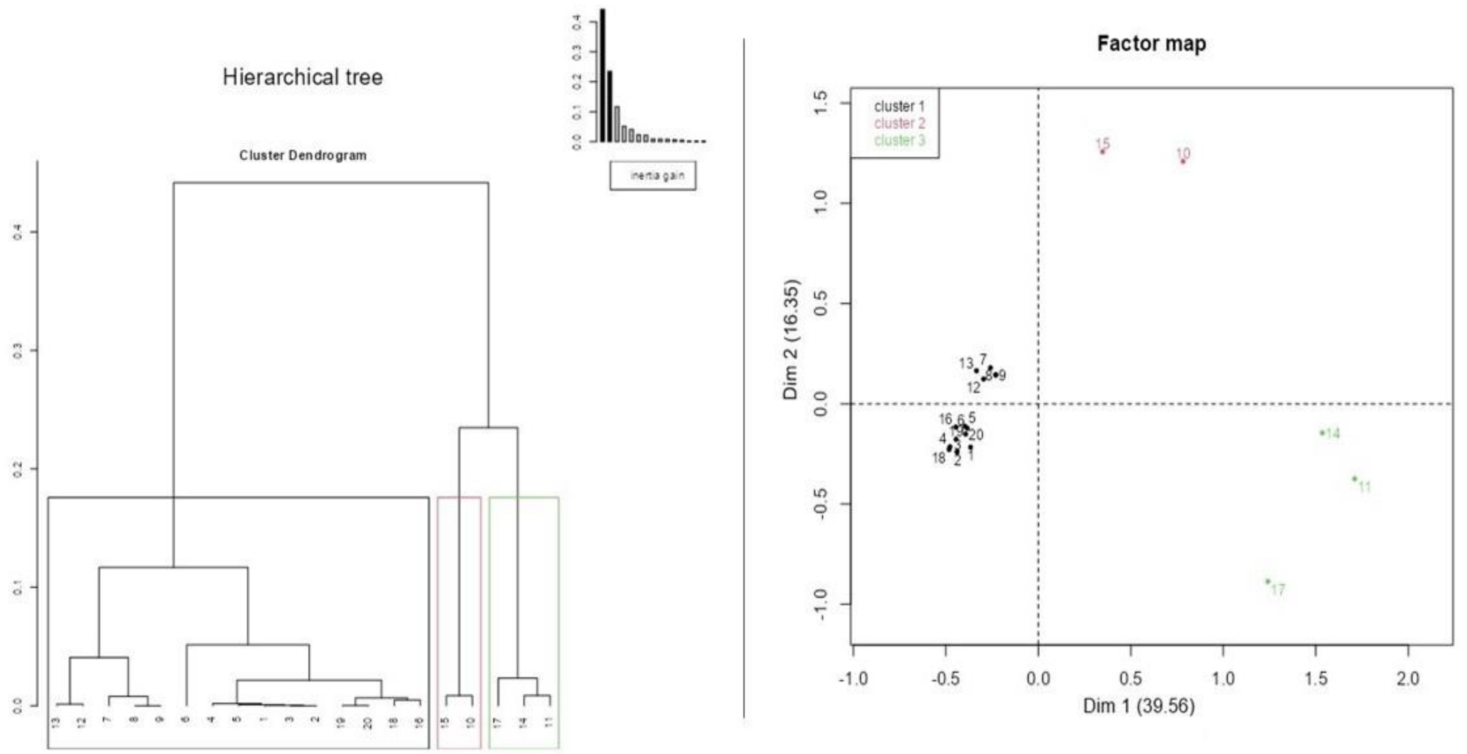

Figure 7: Results of HCPC analysis: representation of individuals in each cluster along dimensions 1 and 2

Table I: Estimates and confidence intervals obtained from the Fisher's exact test

**Variable name**	**Estimate (Confidence Intervals)**	***p*-value**
Crop: maize	9.56 (0.69, 586.77)	0.11
Crop: rice	NA (NA)	NA
Crop: cassava	Inf (3.46, Inf)	0
Crop: sweet potatoes	4.25 (0.32, 252.28)	0.32
Crop: sugar cane	Inf (1.64, Inf)	0.01
Wild fruit: jujube	0.77 (0.05, 8.98)	1
Wild fruit: mokotra	0.77 (0.05, 8.98)	1
Wild fruit: raphia	0.3 (0.01, 4.11)	0.6
Wild fruit: dioscorea	Inf (3.46, Inf)	0
Wild fruit:nuts	NA (NA)	NA
Wild fruit: yam	Inf (3.46, Inf)	0
Domestic fruit: mango	0.1 (0, 1.46)	0.11
Domestic fruit: goayave	0 (0, 116.79)	1
Domestic fruit: gevi	0 (0, 16.83)	1
Domestic: melon	7.16 (0.53, 430.99)	0.13
Domestic fruit: bambara	Inf (0.08, Inf)	0.25
Domestic fruit: papaya	0 (0, 16.83)	1

Distance between forest and villages	0 (0, 0.29)	0
Distance between crop field and village	Inf (1.64, Inf)	0.01
Distance between water source and village	Inf (1.64, Inf)	0.01
Pig farming system: roaming	Inf (7.74, Inf)	0
Pig farming system: confining	0 (0, 0.13)	0
Pig farming system: tieding	0 (0, 0.61)	0.01
Water sources	0 (0, 0.23)	0

Table II: Results of the HCPC analysis: statistical description of clusters

Features of the 3 clusters of fokontany identified through MCA and HCPC in the 20 fokontany examined. The outcomes of the multifactorial analysis highlight in bold the classification of active and supplementary variables that are either positively (v-test>2) or negatively (v-test<-2) linked to the cluster, denoted as: * for a p-value less than 0.05, ** for a *p*-value less than 0.01, and *** for a *p*-value less than 0.001.

**Variables**	**All fokontany (*n*=20)**	**Cluster 1 (*n*=15)**		**Cluster 2 (*n*=2)**		**Cluster 3 (*n*=3)**	
CROPS AND FRUITS							
**Maize**							
Absence	12 (60%)	**11 (73%)**	**	1 (50%)		0 (0%)	
Presence	8 (40%)	4 (27%)	**	1 (50%)		**3 (100%)**	
**Rice**							
Absence	0 (0%)	0 (0%)	*	0 (0%)		0 (0%)	
Presence	20 (100%)	15 (100%)		**2 (100%)**	**	3 (100%)	**
**Cassava**							
Absence	16 (80%)	0 (0%)	*	1 (50%)	**	0 (0%)	*
Presence	4 (20%)	**15 (100%)**	**	1 (50%)		3 (100%)	*
**Sweet potatoes**							
Absence	9 (45%)	8 (53%)	***	1 (50%)	***	0 (0%)	*
Presence	11 (55%)	**7 (47%)**	***	1 (50%)	**	**3 (100%)**	***
**Sugarcane**							
Absence	17 (85%)	8 (53%)	***	**2 (100%)**	***	0 (0%)	***
Presence	3 (15%)	**7 (47%)**	***	0 (0%)	***	**3 (100%)**	***
**Jujube**							
Absence	11 (55%)	8 (53%)	**	**2 (100%)**	***	1 (33%)	**
Presence	9 (45%)	**7 (47%)**	**	0 (0%)	***	**2 (67%)**	**
**Mokotro**							

Absence	11 (55%)	8 (53%)	**	0 (0%)		1 (33%)	*
Presence	9 (45%)	**7 (47%)**	**	**2 (0%)**	*	2 (67%)	*
**Raphia**							
Absence	12 (60%)	8 (53%)	**	0 (0%)	*	1 (33%)	
Presence	8 (40%)	**7 (47%)**	**	**2 (0%)**	*	2 (67%)	**
**Dioscorea**							
Absence	16 (80%)	0 (0%)	***	0 (0%)		1 (33%)	
Presence	4 (20%)	15 (100%)	**	**2 (0%)**	**	2 (67%)	
**Nuts**							
Absence	20 (100%)	15 (100%)	***	2 (100%)	***	3 (100%)	***
Presence	0 (0%)	0 (0%)	**	0 (0%)		0 (0%)	
**Yam**							
Absence	16 (80%)	15 (100%)	**	2 (100%)	*	1 (33%)	*
Presence	4 (20%)	**0 (0%)**	***	0 (0%)	*	**2 (67%)**	*
**Mango**							
Absence	8 (40%)	11 (73%)	**	0 (0%)	*	2 (67%)	*
Presence	12 (60%)	**4 (27%)**	**	0 (0%)	**	1 (33%)	**
**Goayave**							
Absence	19 (95%)	14 (93%)	**	2 (100%)		**3 (100%)**	**
Presence	1 (5%)	**1 (7%)**	**	0 (0%)		0 (0%)	*
**Gevi**							
Absence	18 (90%)	13 (87%)	*	2 (100%)	*	**3 (100%)**	***
Presence	2 (10%)	**2 (13%)**	***	0 (0%)	**	0 (0%)	
**Melon**							
Absence	11 (55%)	10 (67%)	**	0 (0%)	*	2 (67%)	*
Presence	9 (45%)	5 (33%)	**	2 (100%)	***	1 (33%)	
**Watermelon**							
Absence	11 (55%)	0 (0%)	**	0 (0%)	*	1 (33%)	*
Presence	9 (45%)	15 (100%)	**	2 (100%)	*	2 (67%)	**
**Bambara**							
Absence	19 (95%)	15 (100%)	***	2 (100%)	***	2 (67%)	*
Presence	1 (5%)	0 (0%)	**	0 (0%)		1 (33%)	*
**Papaya**							
Absence	18 (90%)	13 (87%)	**	2 (100%)	**	3 (100%)	*
Presence	2 (10%)	**2 (13%)**	**	0 (0%)		0 (0%)	***
GEOGRAPHICAL FEATURES							
**Water points**							
Standpipe	14 (70%)	**14 (93%)**	**	0 (0%)		0 (0%)	***
River & lake	3 (15%)	0 (0%)	*	0 (0%)		**3 (100%)**	***
River	3 (15%)	1 (7%)	**	**2 (100%)**		0 (0%)	
**Distance between village and forest**							
Less 500m	4 (20%)	0 (0%)	***	1 (50%)		**3 (100%)**	***
More 500m	16 (80%)	**15 (100%)**	***	1 (50%)	**	0 (0%)	**

**Distance between village and crop field**							
Less 500m	17 (85%)	**15 (100%)**	***	2 (100%)	*	0 (0%)	*
More 500m	3 (15%)	0 (0%)	***	0 (0%)	*	**3 (100%)**	*****
**Distance between village and water point**							
Less 500m	17 (85%)	**15 (100%)**	***	2 (100%)	**	0 (0%)	***
More 500m	3 (15%)	0 (0%)	***	0 (0%)	**	**3 (100%)**	******
BUSHPIG POPULATION							
**Bushpig population**							
Decrease	12 (60%)	**11 (73%)**	***	0 (0%)		0 (0%)	***
Stable	6 (30%)	1 (7%)		0 (0%)		0 (0%)	
Increase	2 (10%)	3 (20%)	***	2 (100%)		**3 (100%)**	*******
Average number of months of bushpig presence	4.8	3.8	*	6.5	*	8.3	*
**Season of bushpig presence**							
Dry season	16 (80%)	**15 (100%)**	**	1 (50%)	**	0 (0%)	***
Rainy & dry seasons	4 (20%)	0 (0%)	**	1 (50%)		*****	*******
OBSERVATION OF HYBRID & INTERACTION							
**Observation of hybrids in the fokontany**							
Yes	7 (35%)	3 (20%)	***	2 (100%)		**3 (100%)**	******
No	13 (65%)	**12 (80%)**	***	0 (0%)		0 (0%)	*
**Observation of interaction**							
None	15 (75%)	15 (100%)	***	0 (0%)	***	0 (0%)	*
Direct interaction	2 (10%)	0 (0%)		0 (0%)		**2 (67%)**	*******
Indirect interaction	3 (15%)	0 (0%)	***	**2 (100%)**	*******	1 (33%)	*
**Frequence of interaction observed per year**							
>10	3 (15%)	NA		0 (0%)		3 (100%)	
6 to 10	2 (10%)	**NA**		**2 (100%)**		0 (0%)	
CHARACTERISTICS OF PIG FARMING							
**Pig farming system**							
50% roamed & 50%tied	2 (10%)	0 (0%)	***	**2 (100%)**		0 (0%)	
75% confined & 25% tied	15 (75%)	**15 (100%)**		0 (0%)		0 (0%)	*
All pigs roamed	3 (15%)	0 (0%)	***	0 (0%)		**3 (100%)**	
**Number of pigs per farm**							
1 to 5	17 (85%)	**15 (100%)**	***	2 (100%)		0 (0%)	
More 5	3 (15ù)	0 (0%)	***	0 (0%)		**3 (100%)**	*******

Fokontany typology

Data from responses to the interview guide and from proportional piling were integrated with hierarchical classification to enable identification of groups of fokontany with similar environmental conditions to define the variables that influenced the interactions (Table III).

This first step consisted of a multiple correspondence analysis (MCA) followed by hierarchical clustering on principal components (HCPC). This approach that had reported incursions or interactions between bushpigs and domestic pigs.
In this way, each village was classified in a group according to the pig-farming system, the agricultural and environmental conditions of the area, and the participants' perception of other indicators. These indicators can highlight and confirm interactions between bushpigs and domestic pigs, such as the presence of hybrids and the number of months bushpigs are present in the village.

Dimension 1 was mainly influenced by factors related to the presence of cassava, the months of bushpig visits, the distance between the village and the forest, the season of bushpig visits to crop fields, and the number of crops in each village (Figure 7). Dimension 2 was influenced by the presence of the roamed pig (50%) and pig tethered (50%) during rainy season, the presence of melon and watermelon for domestic consumption around the village (and the presence of wild fruits on local palm trees such as raffia (*Raphia farinifera*), mokotra (*Strichnos spinosa)*, jujube (*Ziziphus jujuba*) and the presence of river for water supply. Dimension 3 encompassed the most important factors related to the number of domestic fruits around the village and the water supply points in the village (river). Crops (number, type) strongly influence dimension 4, while dimension 5 was influenced by the dynamic of the bushpig population (i.e., stable, decreasing, increasing).

The MCA/HCPC approach allowed the identification of three types of fokontany (Figure 7). The most represented type of locality (75%) was cluster 1, corresponding to villages located more than 500 m from the forest, less than 500 m from crop fields and water points consisting exclusively of wells (Table V in manuscript). In this cluster, the visit of bushpigs occurred particularly during the dry season and they came on average one to four months per year. A decrease of the bushpig population in the last two years was reported in most of these villages (74%). The presence of hybrids was reported in only 14% of fokontany in this group. No wild fruits (mokotra, raffia, jujube) or sugar cane were growing in this group of localities. Sweet potato was the only tuber grown in these fokontany. This cluster consisted of 5 fokontany within the Boeny region (fokontany n° 1, 5, 19, 18 and 20) and 10 fokontany in the Menabe region (fokontany n° 2, 3, 4, 6, 7, 8, 9, 12, 13 and 16). In this group, despite the visits of bushpigs, there was no reported interaction between bushpig and domestic pig. In this group, the majority of the animals (almost 75%) were kept in confinement throughout the year, and about 25% were tethered during the rainy season which coincides sowing period. They were fed a specific diet consisting of purchased feed from depots and agricultural by-products. On average, each farm had between one and five pigs.

Cluster 3 came in second place (15%), this group was composed of fokontany located less than 500 m from the forest and 500 m from crop fields, mainly composed of maize, yam, sugar cane and cassava (Table *II*). Three fokontany made up this cluster, namely fokontany n° 11 and 14 in Menabe region, and fokontany n° 17 in Boeny region. The water points used by households and for watering domestic animals were located more than 500 m from the village and consisted mainly of lake and river. Wild fruits growing around these fokontany included jujube, raffia fruit and mokotra. Several tubers were planted and grew naturally there such as yam, cassava, sweet potato, and taro. An increase in the population of bushpig compared to two years ago and the presence of hybrid pigs were reported in these fokontany. Bushpig visits occurred during all seasons with an average presence of over 7 months throughout the year. Direct and indirect interactions were observed in the fokontany forming this group. They usually occurred more than ten times a year. One of the causes of agonistic interactions described was male bushpigs trying to mate with domestic sows in heat and fighting with boars. The presence of hybrids was reported in the fokontany encompassing this group. Other reported causes also included simple

fights between the two species. These interactions occurred mainly at night in crop fields. Sharing space for watering and feeding was also reported as another form of direct interaction. This trophic interaction occurred exclusively at the edge of water sources, fruit trees and in crop fields. The pigs in the fokontany of this group roam throughout the year. The animals were occasionally fed with agricultural by-products (rice bran, corn bran) and were free to choose their food. On each farm, the number of pigs ranged from 5 to 10.

Cluster 2 was in last place (10%) and was exclusively composed of two fokontany, n° 10 and 15, both located in the Menabe regions. These fokontany presented a similarity to cluster 3 in terms of distance separating villages from water points, crop fields and the forest (Table *II*). The water sources used by the villagers in this case were exclusively rivers. In these fokontany, only one kind of wild fruit (jujube) was present. Bushpigs visited the villages on average 4-7 months per year. In this group, indirect interaction was the only type of interaction reported. Interactions were detected 6 to 10 times per year. However, there are hybrids in these fokontany. More than half of the pigs raised in the fokontany from this group roamed freely throughout the year. The other half (50%) were tethered to avoid crop damage during the rainy season. The animals were fed mainly on agricultural by-products (rice bran, corn bran) and kitchen waste. The number of pigs per farm varied from 1 to 5.

Table III: List of the 17 Qualitative variables considered in the MCA Hierarchical Classification on Principal Components (HCPC)

**Agricultural context**	
Wild fruit	Absent/ free text
Number of wild fruits in each village	0/1/<2
Crop: tuber	sweet potato/cassava/yam/taro
Crop: sugar cane	Absent/present
Number of crops in each village	0_1/<2_5/<5
**Environmental context**	
Distance village- water points	Less 500m/ More 500m
Water points	Public wells/River/River and lake
Distance village-forest	Less 500m/ More 500m
Distance village-crop fields	Less 500m/ More 500m
**Dynamic of bushpig around the village**	
Number of months of bushpig presence around the village	<1-4 months/<4-7 months /<7 months
Population of bushpigs	Increase/Stable/Decrease
**Characteristics of interactions reported**	
Hybrid observation	Yes/No
Observation of interactions in the last 12 months	Yes/No
Types of interactions observed	None/Indirect interaction/Direct interaction
Frequency of interactions	At least once per month/More than once per week
Number of times per year	06_10/>10
**Farm characteristics**	
Pig farming system	pigs confined/pigs tied/pigs roamed

<u>References cited</u>

1. Kassambara, A. ACM - Analyse des Correspondances Multiples avec R: L'Essentiel. *STHDA* http://www.sthda.com/french/articles/38-methodes-des-composantes-principales-dans-r-guide-pratique/75-acm-analyse-des-correspondances-multiples-avec-r-l-essentiel/ (2017).
2. Kassambara, A. HCPC - Hierarchical Clustering on Principal Components: Essentials - Articles - STHDA. *Statistical tools for high-throughput data analysis* http://www.sthda.com/english/articles/31-principal-component-methods-in-r-practical-guide/117-hcpc-hierarchical-clustering-on-principal-components-essentials (2017).
3. Tam, T. A Visualization and Analysis of Categorical Data Using R and SOCR. (2012).

Appendix 2: List of digited maps

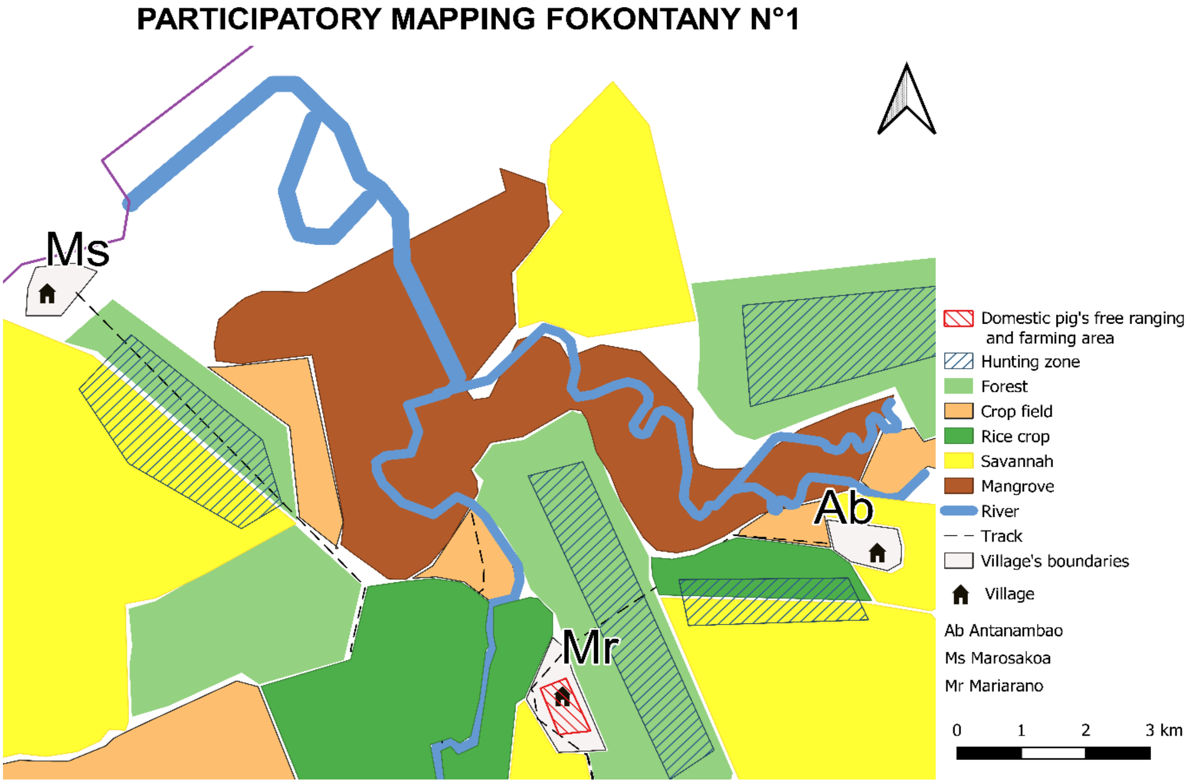

Figure 8: Participatory mapping fokontany n°1. Paperboard map (69x99 cm) containing main geographical features (main roads, lakes and rivers) available on https://openstreetmap.org/ was used as basemap. Using the basemap, participants characterised their territories, a physical copy of the map was scanned and processed to create georeferencing using ground control points through spline or first-order polynomial transformations. The resulting georeferenced map was then exported and processed into QGIS 3.10, a Coruña free open source software http://qgis.org/ in raster format. Participants reviewed and corrected the digitized paper maps.

PARTICIPATORY MAPPING FOKONTANY N°2

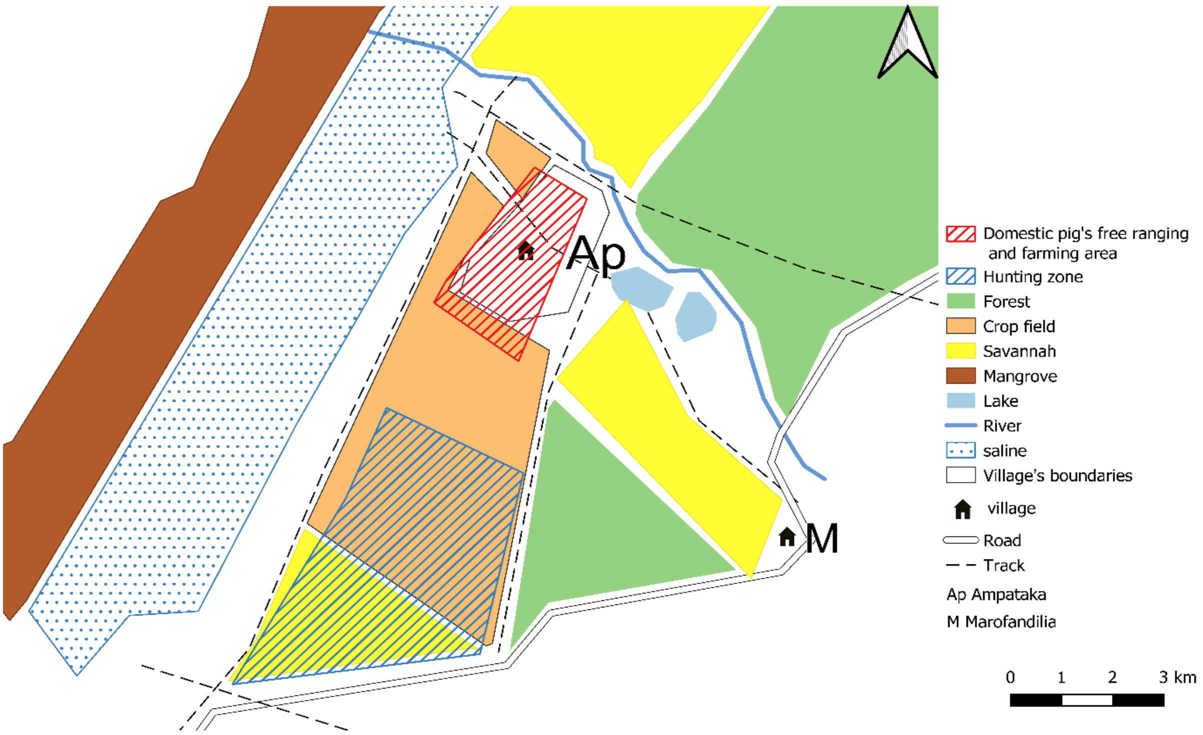

Figure 9: Participatory mapping fokontany n°2. Paperboard map (69x99 cm) containing main geographical features (main roads, lakes and rivers) available on https://openstreetmap.org/ was used as basemap. Using the basemap, participants characterised their territories, a physical copy of the map was scanned and processed to create georeferencing using ground control points through spline or first-order polynomial transformations. The resulting georeferenced map was then exported and processed into QGIS 3.10, a Coruña free open source software http://qgis.org/ in raster format. Participants reviewed and corrected the digitized paper maps.

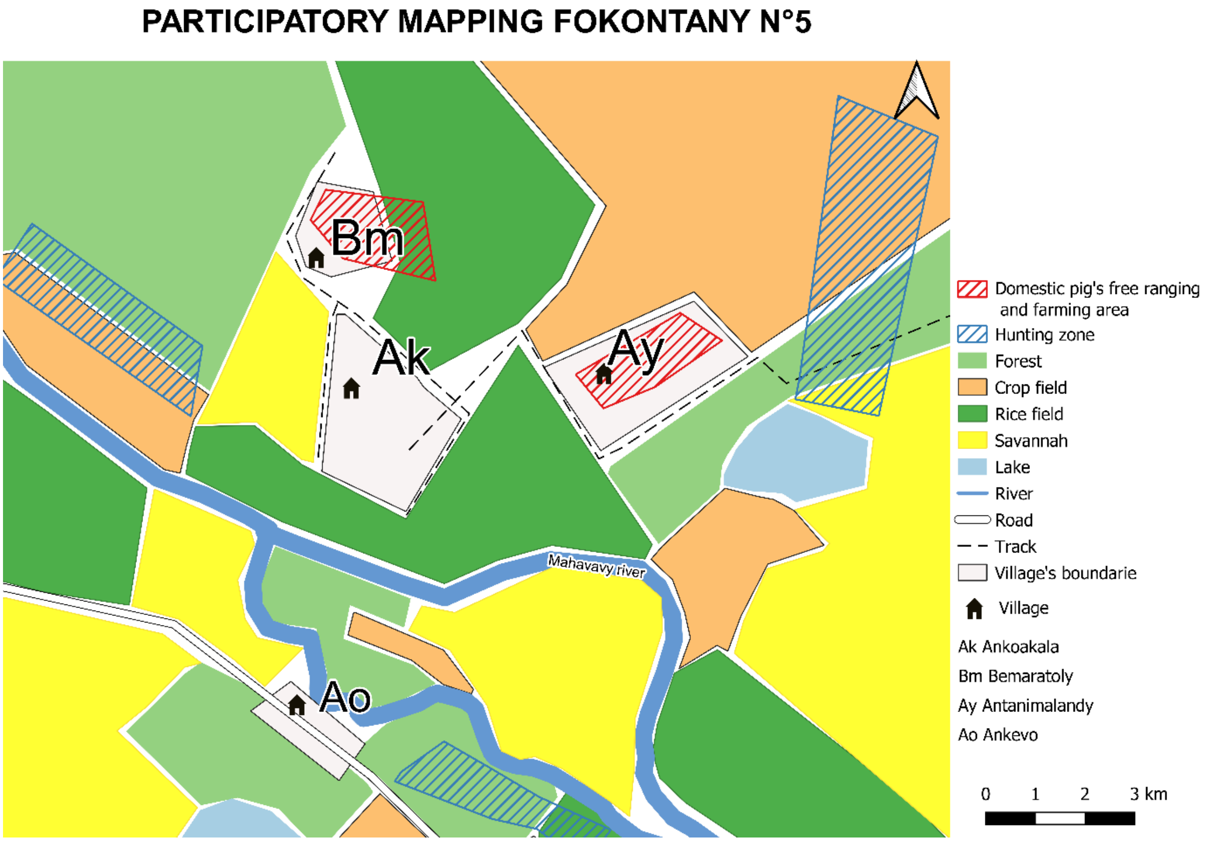

Figure 10: Participatory mapping fokontany n°5. Paperboard map (69x99 cm) containing main geographical features (main roads, lakes and rivers) available on https://openstreetmap.org/ was used as basemap. Using the basemap, participants characterised their territories, a physical copy of the map was scanned and processed to create georeferencing using ground control points through spline or first-order polynomial transformations. The resulting georeferenced map was then exported and processed into QGIS 3.10, a Coruña free open source software http://qgis.org/ in raster format. Participants reviewed and corrected the digitized paper maps.

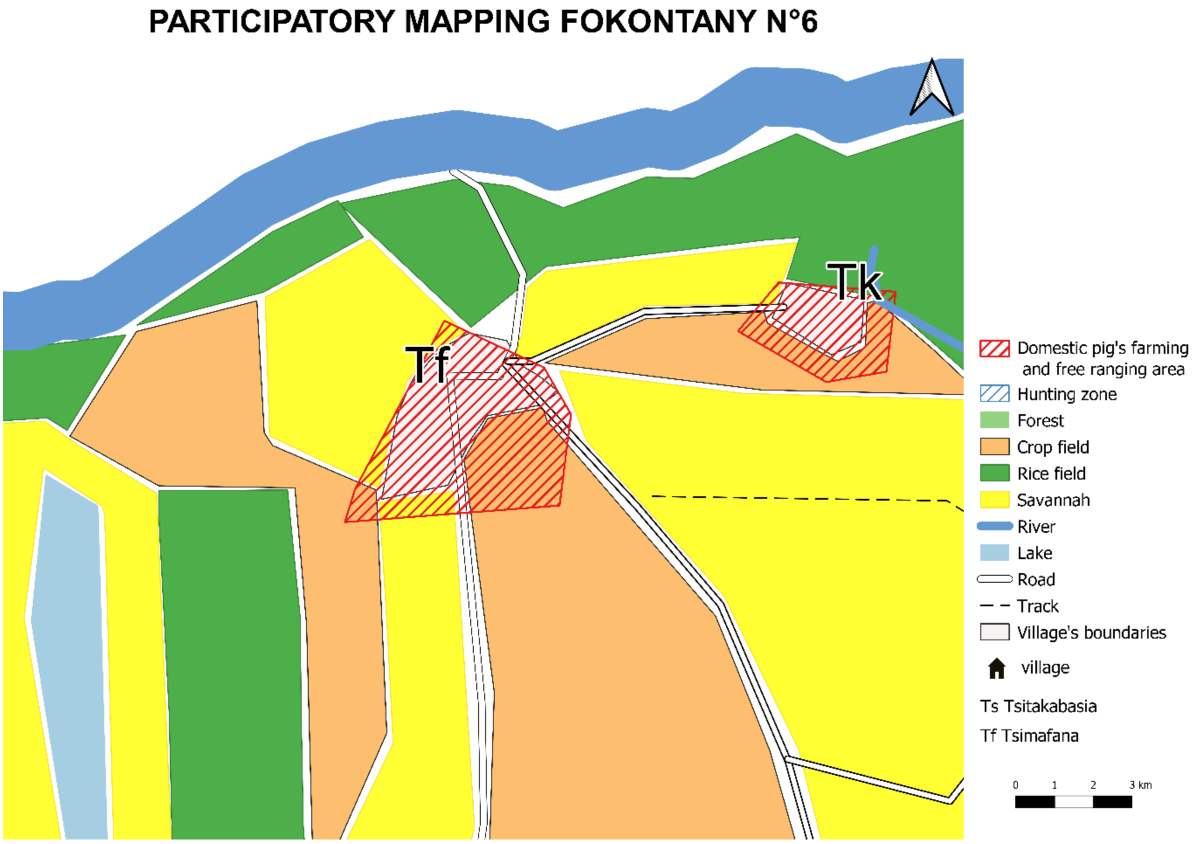

Figure 11: Participatory mapping fokontany n°6. Paperboard map (69x99 cm) containing main geographical features (main roads, lakes and rivers) available on https://openstreetmap.org/ was used as basemap. Using the basemap, participants characterised their territories, a physical copy of the map was scanned and processed to create georeferencing using ground control points through spline or first-order polynomial transformations. The resulting georeferenced map was then exported and processed into QGIS 3.10, a Coruña free open source software http://qgis.org/ in raster format. Participants reviewed and corrected the digitized paper maps.

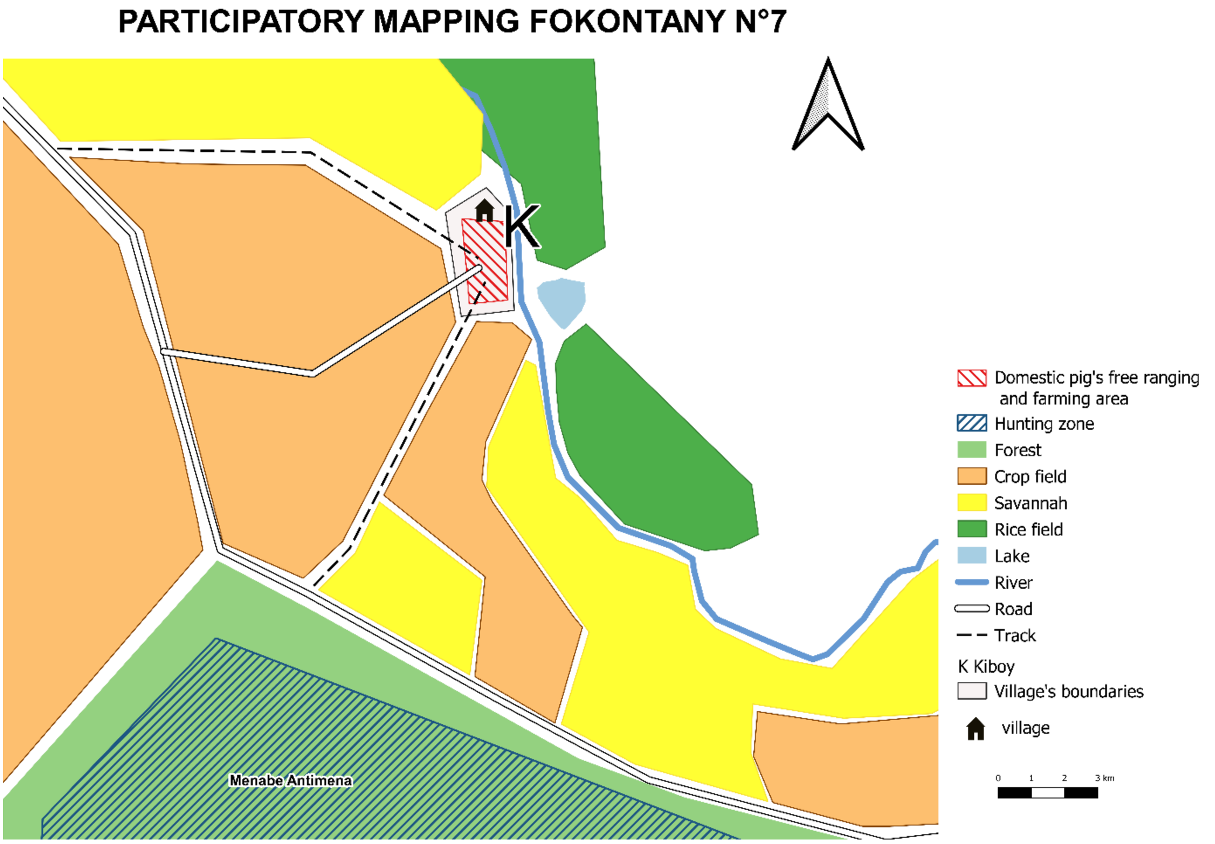

Figure 12: Participatory mapping n°7. Paperboard map (69x99 cm) containing main geographical features (main roads, lakes and rivers) available on https://openstreetmap.org/ was used as basemap. Using the basemap, participants characterised their territories, a physical copy of the map was scanned and processed to create georeferencing using ground control points through spline or first-order polynomial transformations. The resulting georeferenced map was then exported and processed into QGIS 3.10, a Coruña free open source software http://qgis.org/ in raster format. Participants reviewed and corrected the digitized paper maps.

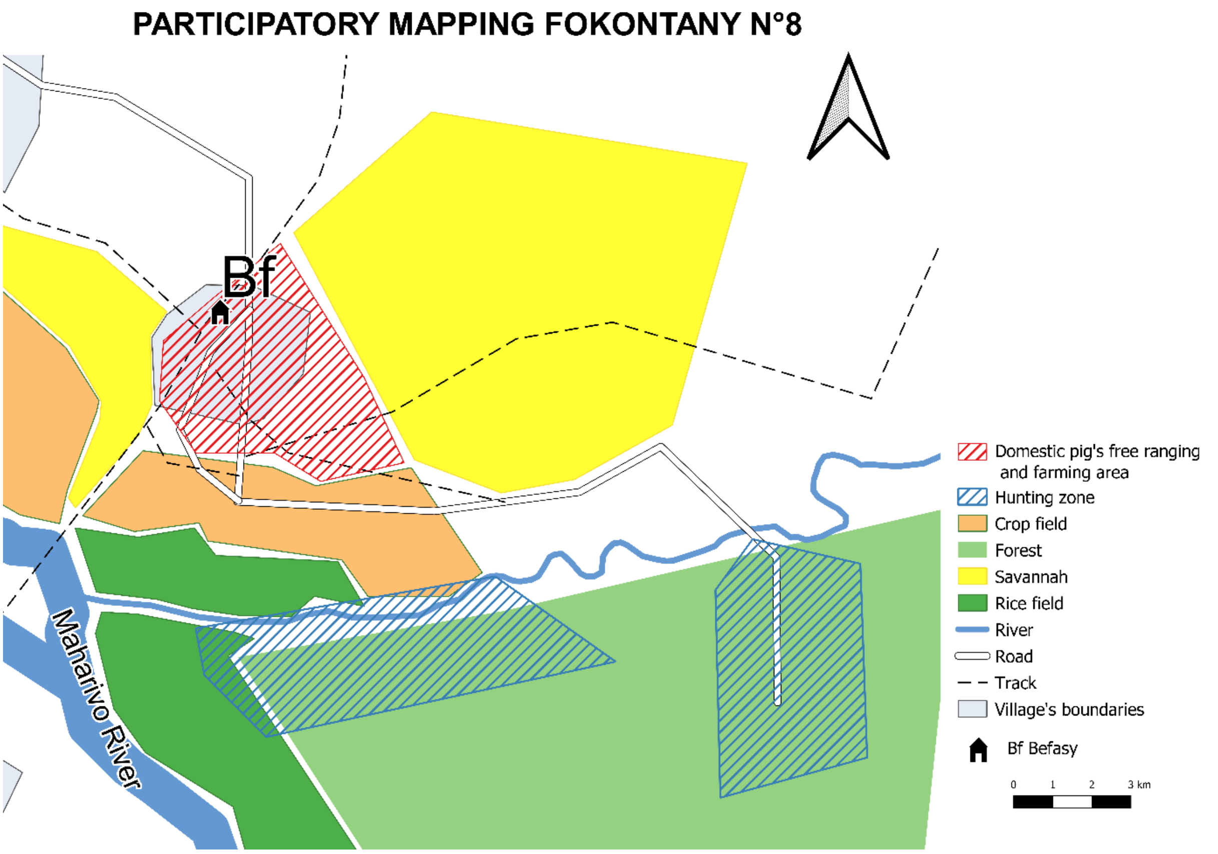

Figure 13: Participatory mapping fokontany n°8. Paperboard map (69x99 cm) containing main geographical features (main roads, lakes and rivers) available on https://openstreetmap.org/ was used as basemap. Using the basemap, participants characterised their territories, a physical copy of the map was scanned and processed to create georeferencing using ground control points through spline or first-order polynomial transformations. The resulting georeferenced map was then exported and processed into QGIS 3.10, a Coruña free open source software http://qgis.org/ in raster format. Participants reviewed and corrected the digitized paper maps.

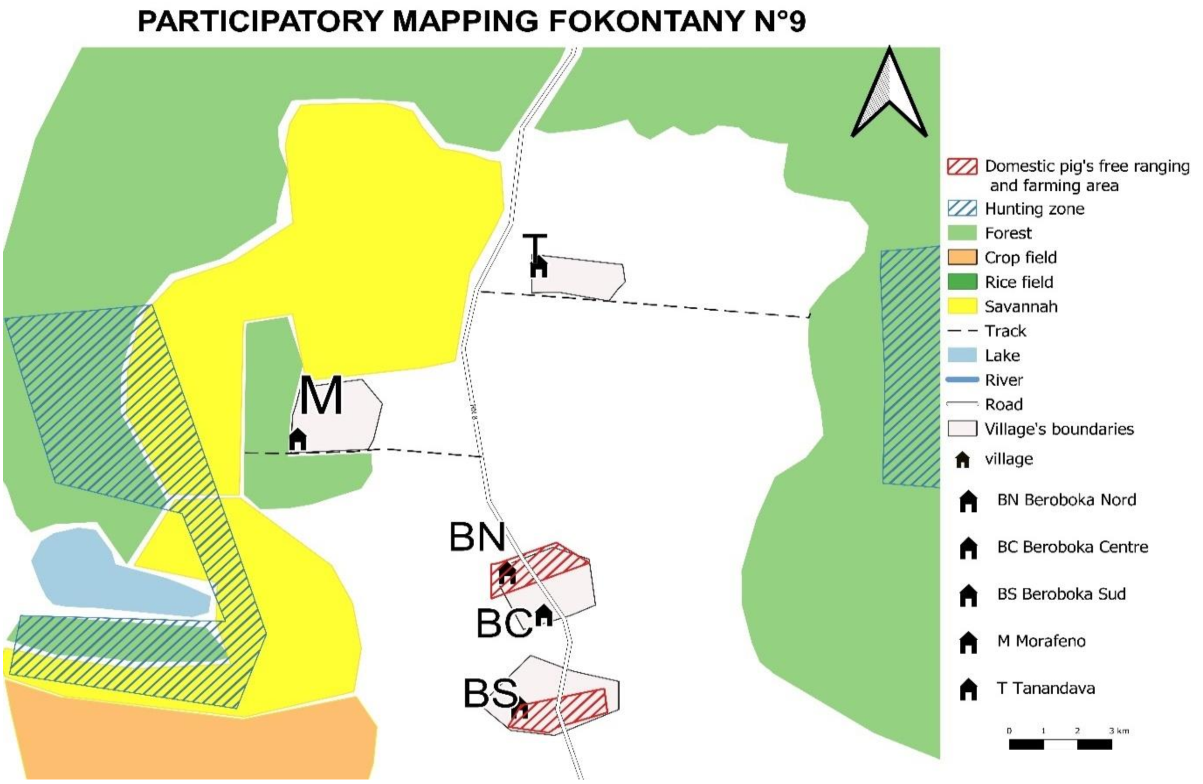

Figure 14: Participatory mapping n° 9. Paperboard map (69x99 cm) containing main geographical features (main roads, lakes and rivers) available on https://openstreetmap.org/ was used as basemap. Using the basemap, participants characterised their territories, a physical copy of the map was scanned and processed to create georeferencing using ground control points through spline or first-order polynomial transformations. The resulting georeferenced map was then exported and processed into QGIS 3.10, a Coruña free open source software http://qgis.org/ in raster format. Participants reviewed and corrected the digitized paper maps.

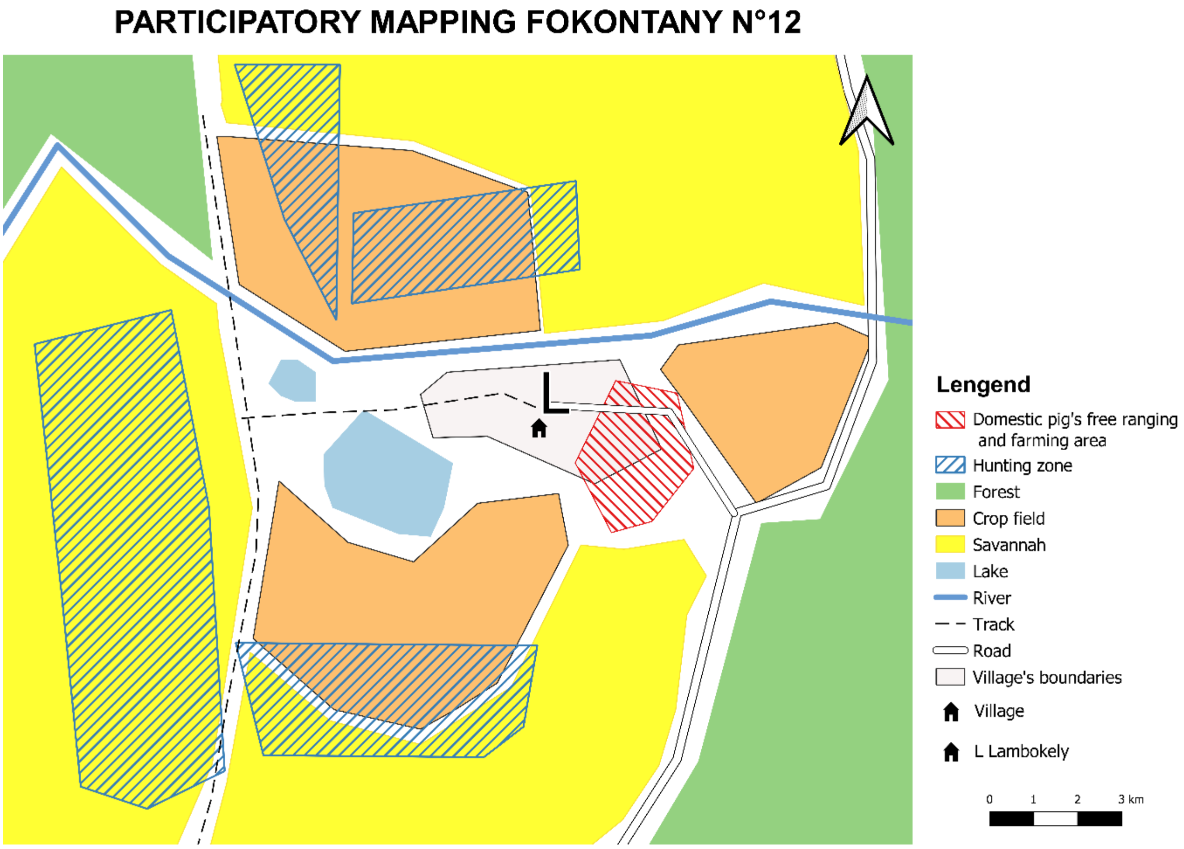

Figure 15: Participatory mapping n°12. Paperboard map (69x99 cm) containing main geographical features (main roads, lakes and rivers) available on https://openstreetmap.org/ was used as basemap. Using the basemap, participants characterised their territories, a physical copy of the map was scanned and processed to create georeferencing using ground control points through spline or first-order polynomial transformations. The resulting georeferenced map was then exported and processed into QGIS 3.10, a Coruña free open source software http://qgis.org/ in raster format. Participants reviewed and corrected the digitized paper maps.

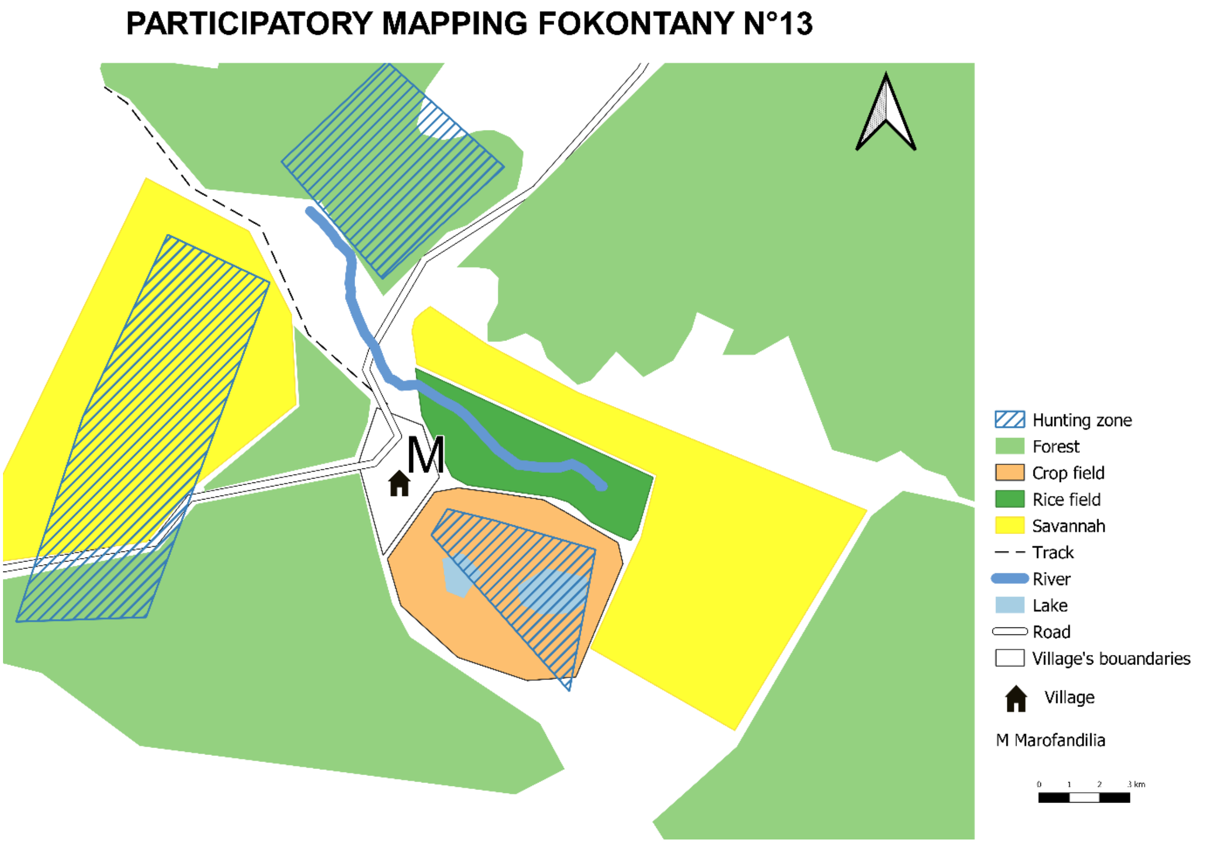

Figure 16: Participatory mapping n°13. Paperboard map (69x99 cm) containing main geographical features (main roads, lakes and rivers) available on https://openstreetmap.org/ was used as basemap. Using the basemap, participants characterised their territories, a physical copy of the map was scanned and processed to create georeferencing using ground control points through spline or first-order polynomial transformations. The resulting georeferenced map was then exported and processed into QGIS 3.10, a Coruña free open source software http://qgis.org/ in raster format. Participants reviewed and corrected the digitized paper maps.

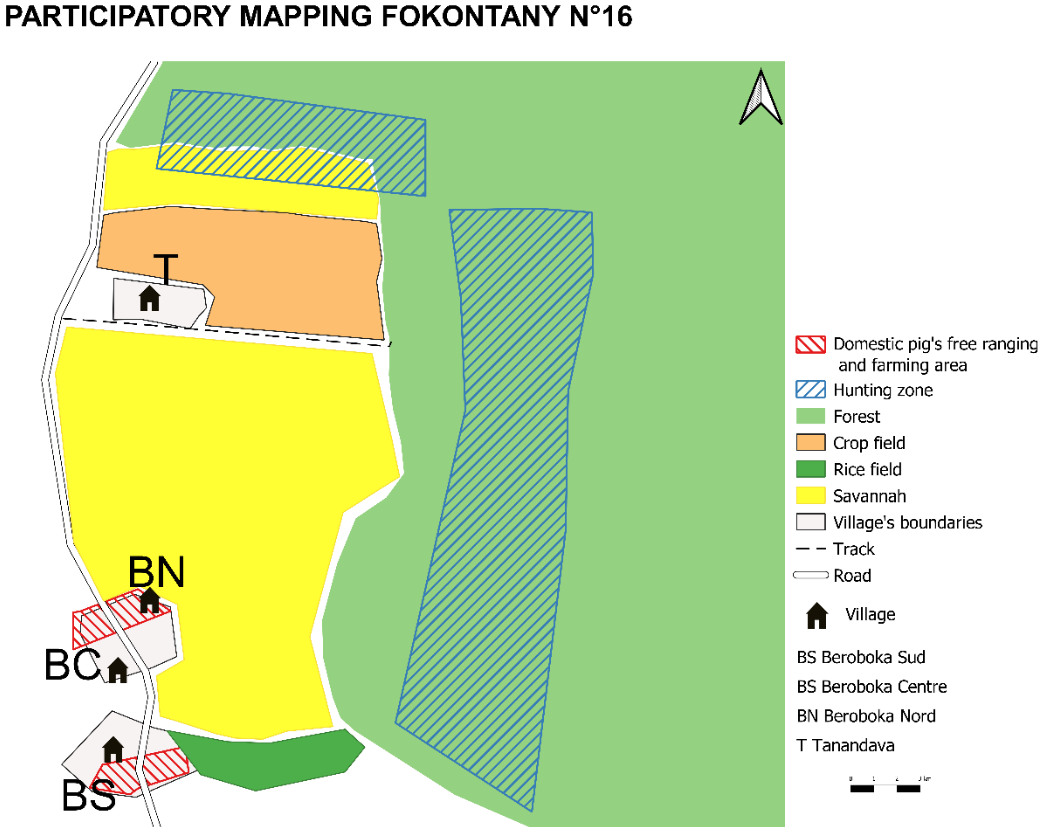

Figure 17: Participatory mapping n°16. Paperboard map (69x99 cm) containing main geographical features (main roads, lakes and rivers) available on https://openstreetmap.org/ was used as basemap. Using the basemap, participants characterised their territories, a physical copy of the map was scanned and processed to create georeferencing using ground control points through spline or first-order polynomial transformations. The resulting georeferenced map was then exported and processed into QGIS 3.10, a Coruña free open source software http://qgis.org/ in raster format. Participants reviewed and corrected the digitized paper maps.

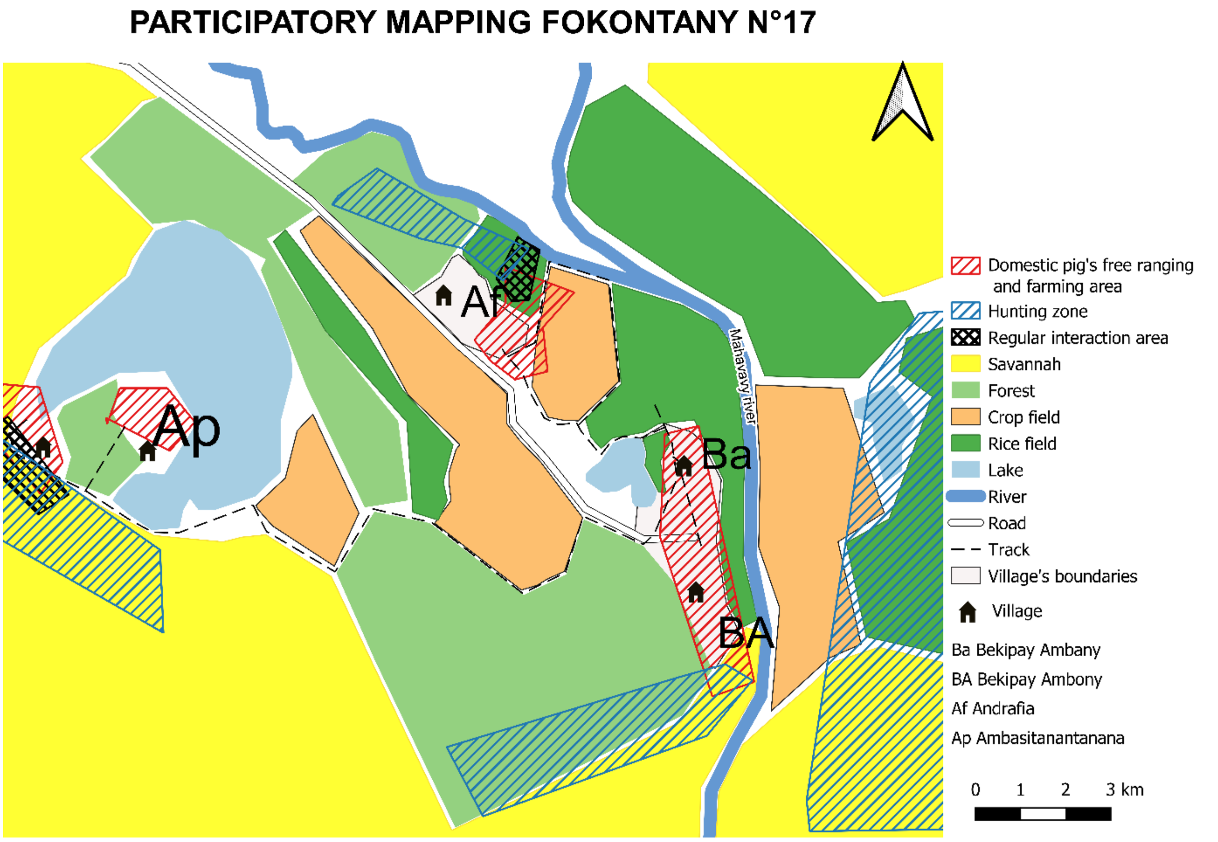

Figure 18: Participatory mapping n°17. Paperboard map (69x99 cm) containing main geographical features (main roads, lakes and rivers) available on https://openstreetmap.org/ was used as basemap. Using the basemap, participants characterised their territories, a physical copy of the map was scanned and processed to create georeferencing using ground control points through spline or first-order polynomial transformations. The resulting georeferenced map was then exported and processed into QGIS 3.10, a Coruña free open source software http://qgis.org/ in raster format. Participants reviewed and corrected the digitized paper maps.

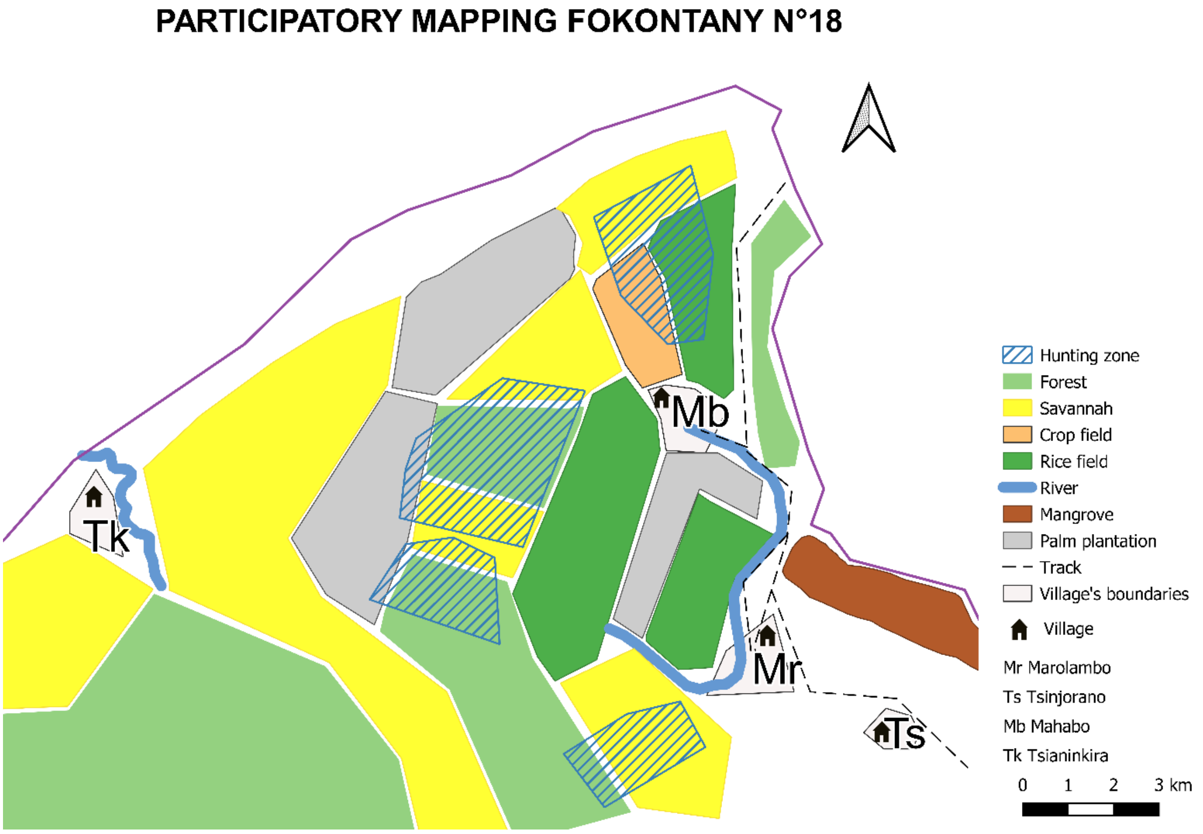

Figure 19: Participatory mapping n°18. Paperboard map (69x99 cm) containing main geographical features (main roads, lakes and rivers) available on https://openstreetmap.org/ was used as basemap. Using the basemap, participants characterised their territories, a physical copy of the map was scanned and processed to create georeferencing using ground control points through spline or first-order polynomial transformations. The resulting georeferenced map was then exported and processed into QGIS 3.10, a Coruña free open source software http://qgis.org/ in raster format. Participants reviewed and corrected the digitized paper maps.

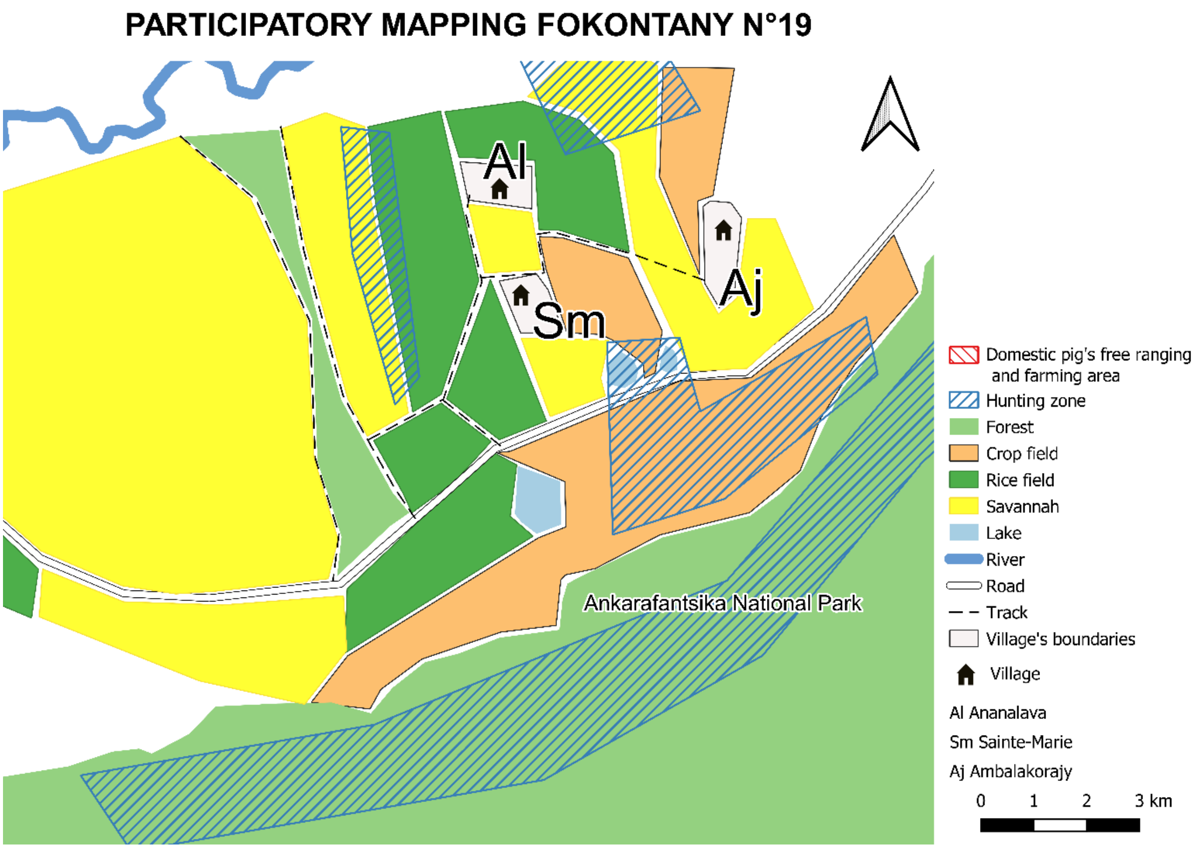

Figure 20: Participatory mapping n°19. Paperboard map (69x99 cm) containing main geographical features (main roads, lakes and rivers) available on https://openstreetmap.org/ was used as basemap. Using the basemap, participants characterised their territories, a physical copy of the map was scanned and processed to create georeferencing using ground control points through spline or first-order polynomial transformations. The resulting georeferenced map was then exported and processed into QGIS 3.10, a Coruña free open source software http://qgis.org/ in raster format. Participants reviewed and corrected the digitized paper maps.

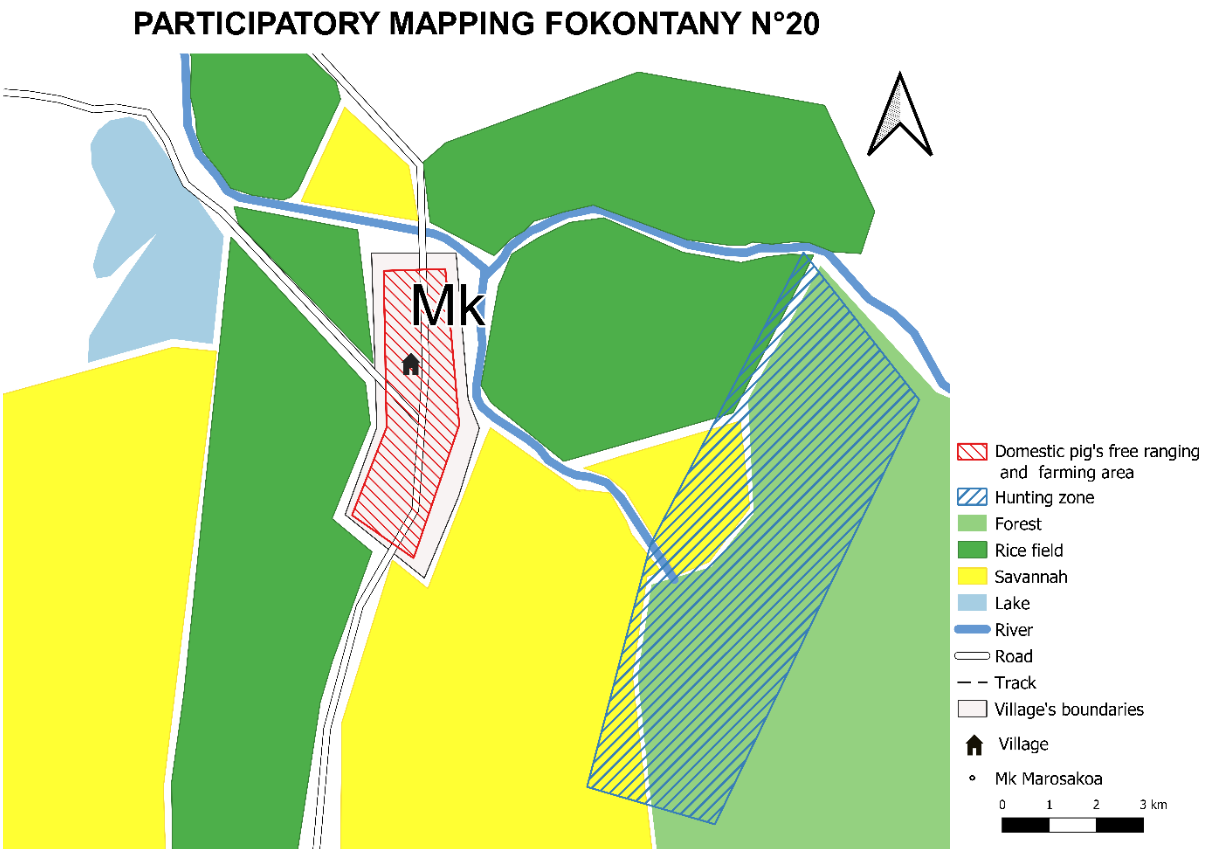

Figure 21: Participatory mapping n°20. Paperboard map (69x99 cm) containing main geographical features (main roads, lakes and rivers) available on https://openstreetmap.org/ was used as basemap. Using the basemap, participants characterised their territories, a physical copy of the map was scanned and processed to create georeferencing using ground control points through spline or first-order polynomial transformations. The resulting georeferenced map was then exported and processed into QGIS 3.10, a Coruña free open source software http://qgis.org/ in raster format. Participants reviewed and corrected the digitized paper maps.

Appendix 3: Questionnaire

Questionnaire n°…………………

Fanontaniana laharana faha:……………………………………………………………………

<u>Part I: Momba ilay toerana</u> <u>(Site description)</u>

1 **Daty** (Date):
2 **Mpanentana** (Facilitator):
3 **Mpandray an-tsoratra** (Note-taker):
4 **Ora nanombohana**: ……. **Ora namaranana**: ………………………………
Start time: Finish time:
5 **Disitirika** (District):
6 **Kaominina** (Commune):
7 **Fokontany**:
8 **Toerana** (Location):
9 **Isan'ny mpandray anjara** (Nb Participants):
10 **lahy/vavy** (Men/Women):

a. Fanadihadiana ny mponina
(Getting to know the community)

1 Firy ny isan'ireo mpiompy/mpamboly/mpihaza ?
How many pig farmer/crop farmer/hunter?

Part II: Fambolena/rano Crop/water source Description

a- Fambolena (crop)

Cereals (Voamaina)	Fruits (Voankoazo)	Tuber (haninkotrana)	Hafa (other)

b- vanin'andro
(Seasonal calendar) *CHECK BACK

c- Rano ampiasaina (Water source):

Lava-drano/paompy (Well)	Matsabory/Farihy (Lake)	Renirano (River)	Hafa (other)

Part III: Toerana misy ilay fokontany (Location of the fokontany)

Halaviran'ny tanàna sy ny atiala (Distance between village and forest)	Halaviran'ny tanàna sy ny tanimboly (Distance between village and crop field)	Halaviran'ny tanàna sy ny fakana rano (Distance between village and water point)

Part IV: Momba ny lambo (Bushpig population)

1. **Mandalo manodidina ny tanàna ve ny lambodia?** (Do bushpigs visit around the village?)
2. **Mihena ve sa mitombo sa mitovy ny isan'ny lambodia tato anatin'ny heritaona?** (has the bushpig population decreased, increased or remained the same over the past year?)
3. **Volana inona no mandalo manodidina ny tanàna ny lambodia?** (In which month bushpig visit around village?) (Cf calendar)

Part V: Fifandraisana (Interaction)

i- Fifandraisana mivantana(kisoa sy lambo) Direct interactions

1. **Efa nahita fifandraisana mivantana teo @ kisoa sy lambodia?** (have they observed direct interaction between domestic pig and bushpig)
2.Raha tsy nahita fifandraisana mivantana izy ireo, efa naheno izany tamin'ny olona hafa ve? (If they have not seen a direct interaction, have they ever heard of it from someone else?)
3. **Firy ny isan'ny olona nahita ilay fifandraisana mivantana niseho?** (How many people have seen direct interactions happening?)
4. **Impiry no nahita izany niseho anatin'ny taona iray?** (How many times have you seen it happening)
5. **Vanin'andro inona/Fotoana inona tao anatin'ny taona io fifandraisana mivantana io no niseho?** (In which season/month of year did the direct interaction happen?)
6. **Toy ny ahoana ilay fifandraisana niseho?ohatra, niray, niaraka nisakafo, niaraka nisotro rano, niady?** (Which type of contact happened? For example, they were mating together, or eating together, or drinking together, or fighting).
7. **Nandritry ny hafiriana teo izy ireo no niaraka teo?**(How long did the animals remain together)
8. **Misy kisoa metisy lambo ve eto an-tanàna? (**Is there a hybrid pig in the village?)
9. **Efa naheno olona nahita kisoa metisy lambo teo an-tanàna?** (Heard of hybrids)

ii- Fifandraisana ankolaka (kisoa sy lambo **)** Indirect interaction

1. **Efa nahita fifandraisana ankolaka teo amin'ny lambo sy kisoa ve na mahalala toerana hifampizaran'izy ireo?** (Have they ever observed indirect interaction, is there a place shared by 2 species at different time?)
2. **Adiny firy ny elanelam-potoana andalovan'ny biby iray sy ny manaraka?** (Time interval between two visits for indirect?)

Part VI: Pig farming system

Hanontaniana ny mpandray anjara ny amin'ny fomba fiompiana kisoa misy eo an-toerana. Atao kisarisary boribory izany eo amin'ny tany. Zarain'ny mpanentana voana tsaramaso 100 ireo mpandray anjara, ary anjaran'izy ireo no mametraka izany anatin'ny boribory araka ny fahazon'izy ireo ny fomba fiompiana eo an-tanàna.

Ask the participants how pigs are raised in the Fokontany. Draw circles on the floor according to the pig farming systme listed. The facilitator distributes 100 beans to the participants, who place them in each circle according to their perception of the importance of each farming system in the fokontany.

Firy eo ho eo ny isan'ny kisoa anatin'ny fiompiana iray? (Number of pigs in each farm)

Sakafo omena ny kisoa (feed)

Seasonal calendar<br>**Alimanaka vanim-potoana**	Months (**Volana**)											
Event (**zava-miseho**)	J	F	M	A	M	J	J	A	S	O	N	D
Crop season $^{\Delta}$<br><br>**Vanim-potoana fambolena**												
Fruit season $^{\Delta}$<br>**Vanim-potoanan'ny voankazo**												
Presence of bushpig in the community<br>**Fisian'ny lambo ao an-toerana**												
Direct interaction<br>**Fifandraisana mivantana eo @ lambo sy kisoa**												

Figure 22: Accordance statement

UNDERSTANDING THE INTERACTION BETWEEN DOMESTIC PIG AND BUSHPIG IN RURAL AREA IN MADAGASCAR

Invitation to participate

We are conducting a research project where we investigate the interactions between domestic pigs and bushpigs in order to characterize interaction between those species. Our final aim is to prevent pig diseases in your community and, in this way, to keep your pigs healthier. We are inviting you to participate in this research.
In this research we want to ask you questions about your pigs, pig keeping, crop and bushpigs sighting and bushpigs interactions with your pigs. If there is a question that you do not want to answer, you can say "I do not want to answer that question". This will take about 90 minutes.

Your consent to participate

Your answers and participations are completely confidential and will be released only as summaries in which no individual's answers can be identified. You can choose not to participate and can quit the survey at any time. There will be no penalty or loss of services or benefits if you decide not to take part in the survey.

Organizations involved in this research

This international collaboration includes personnel from CIRAD, FOFIFA-DRZVP (National Centre for Applied Research in Rural Development- Department of Zootechnical Veterinary and Fish Farming Research), National Veterinary Diagnostic Laboratory (LNDV), Eduardo Mondlane University Mozambique, Institute of Agricultural Research of Mozambique, Onderstepoort Veterinary Institute South Africa, University Complutense of Madrid Spain, University of California Davis U.S.A., and University of Pretoria South Africa, and is funded by a NSF USDA EEID grant (Award #: 2019-67015-28981).

We will keep your information private

The information you provide to us will be kept private and confidential. Only the fieldwork leader Rianja Rakotoarivony and senior members of the research team authorized by Rianja will be able to look up your name. When we use the information about you or your pigs in our research reports, we will not use your name or the exact location of your farm.

Benefits and risks to participants

We are doing this research to help the understanding of the interactions that may exist between your domestic pigs and bushpig and the consequences of this with regards to disease transmission and genetic implication. These are big and complicated problems. This research will not provide all the answers that are needed and will not stop the risk of your pigs getting sick.

Contacts for questions or problems about the research

If you have questions about the research after today, you should contact one of these people on the research team:

- Fieldwork leader: Rianja Rakotoarivony
- Fieldwork collaborators: Alpha Andriamahefa, Diana Andria-Mananjara, Mihaja Rakotoarinoro, Herilanto Ramaroson, Modestine Raliniaina, Miatrana Rasamoelina

Many thanks for your participation!!!
